# Identification of common oncogenic and early developmental pathways in the ovarian carcinomas controlling by distinct prognostically significant microRNA subsets

**DOI:** 10.1186/s12864-017-4027-5

**Published:** 2017-10-03

**Authors:** Vladimir A. Kuznetsov, Zhiqun Tang, Anna V. Ivshina

**Affiliations:** 1 Genome and Gene Expression Data Analysis Division, Bioinformatics Institute, A-STAR, 30 Biopolis Street, #07-01 Matrix, Singapore, 138671 Singapore; 2 School of Computer Science and Engineering, Nanyang Technological University, Singapore, 639798 Singapore

**Keywords:** ovarian cancer, microRNA, prognostic signatures, oncogenic pathway, EMT, neurotrophin signaling, progesterone-mediated oocyte maturation, weighted voting grouping, meta-analysis, prognostic biomarker

## Abstract

**Background:**

High-grade serous ovarian carcinoma (HG-SOC) is the dominant tumor histologic type in epithelial ovarian cancers, exhibiting highly aberrant microRNA expression profiles and diverse pathways that collectively determine the disease aggressiveness and clinical outcomes. However, the functional relationships between microRNAs, the common pathways controlled by the microRNAs and their prognostic and therapeutic significance remain poorly understood.

**Methods:**

We investigated the gene expression patterns of microRNAs in the tumors of 582 HG-SOC patients to identify prognosis signatures and pathways controlled by tumor miRNAs. We developed a variable selection and prognostic method, which performs a robust selection of small-sized subsets of the predictive features (e.g., expressed microRNAs) that collectively serves as the biomarkers of cancer risk and progression stratification system, interconnecting these features with common cancer-related pathways.

**Results:**

Across different cohorts, our meta-analysis revealed two robust and unbiased miRNA-based prognostic classifiers. Each classifier reproducibly discriminates HG-SOC patients into high-confidence low-, intermediate- or high-prognostic risk subgroups with essentially different 5-year overall survival rates of 51.6-85%, 20-38.1%, and 0-10%, respectively. Significant correlations of the risk subgroup’s stratification with chemotherapy treatment response were observed. We predicted specific target genes involved in nine cancer-related and two oocyte maturation pathways (neurotrophin and progesterone-mediated oocyte maturation), where each gene can be controlled by more than one miRNA species of the distinct miRNA HG-SOC prognostic classifiers.

**Conclusions:**

We identified robust and reproducible miRNA-based prognostic subsets of the of HG-SOC classifiers. The miRNAs of these classifiers could control nine oncogenic and two developmental pathways, highlighting common underlying pathologic mechanisms and perspective targets for the further development of a personalized prognosis assay(s) and the development of miRNA-interconnected pathway-centric and multi-agent therapeutic intervention.

**Electronic supplementary material:**

The online version of this article (doi:10.1186/s12864-017-4027-5) contains supplementary material, which is available to authorized users.

## Background

Ovarian cancer (OC) is a fatal gynecologic malignancy and is a highly heterogeneous disease comprising many distinct tumor types [1–3]. Serous ovarian cancer (SOC) accounts for 90% of ovarian malignancies and is often diagnosed in advanced stages due to the lack of an effective screening and early malignancy diagnostic method(s). It is also a fact that overall survival (OS) improvements have been difficult to achieve with the existing drugs. The mortality rate for SOC patients has remained consistently high, with a 5-year survival rate of approximately 30% over the past decades [1]. SOC was reported to frequently exhibit high intra-tumor heterogeneity, genomic instability, multiple mutations, stem-cell diversity, and genetically defined disease subgroups. These observations were frequently observed in high-grade serous ovarian cancer (HG-SOC) [4, 5], which consists of multiple and poorly characterized tumor subtypes [4, 6, 7]. Despite fast progress in medicine, technology and our understanding of etiology and patho-biological mechanisms of several cancers, better molecular classification, more accurate personalized prognosis and prediction of the disease risk, and the discovery of more effective and precise therapeutic interventions are urgently needed for HG-SOC.

It is vital to characterize HG-SOC at the molecular level and if possible, to provide a careful classification of the disease subtypes so that the difference among subtypes can be reflected in clinical research design and SOC management. However, clinical and histo-pathological factors present limited abilities in the prediction of SOC patient risk with statistical confidence [8]. For example, the clinical biomarker CA125 has been proposed to stratify ovarian cancer patients [9–11]. However, 20-30% of SOCs do not produce reliably detectable CA125 [12], which therefore cannot serve as an applicable marker for diagnostic of malignancy and prognosis of SOC patients. Survival analysis of some SOC clinical biomarkers, such as CA125, HE4, MYC and P53, have demonstrated that these markers exhibit limited prognosis and prediction capabilities [8].

Several well-organized genome wide studies have sought to identify the molecular subtypes of HG-SOC [8, 13–17]. In ovarian cancer, the Cancer Genome Atlas (TCGA) project revealed four molecular subtypes, including the differentiated, immune-reactive, mesenchymal and proliferative subtypes [14]. The molecular subtypes were suggested based on the cluster analysis of the protein-coding gene expression data. However, there were poor survival differences between the patient subgroups assigned by the four subtypes [8, 14]. A meta-analysis of a large collection of mRNAs unveiled five molecular subtypes grouped onto two marginal overall survival patterns [18]. The Australian ovarian cancer study (AOCS) observed six molecular subtypes using gene expression profiles of EOC samples [13, 19]. The “Classification of Ovarian Cancer” (CLOVAR) survival signature is a prognostic model of HG-SOC classification containing 100 protein-coding genes whose expression were most correlated or anti-correlated with the TCGA cohort patient survival [20] and whose expression stratified patients onto low-, intermediate- and high-risk prognostic subgroups. However, when testing using independent cohort, CLOVAR was unable to discriminate intermediate- and low-risk patient subgroups [20]. Further development of the HG-SOC classifiers considering the disease-associated non-codding for protein regulatory molecules could be important in clinical applications.

MicroRNAs (miRNAs), which are short (approximately 21-22 nucleotides) RNA molecules, regulate gene expression post-transcriptionally by targeting mRNAs for cleavage or translational repression. The miRNAs thought to hold great promise as prognostic and predictive biomarkers [21–23]. The miRNAs could realize it therapeutic potential either through regulating the expression of key genes, pathways or through being targeted themselves by anti-miRNAs agents. Because a single miRNA has multiple target genes, the miRNAs can regulate both oncogene and tumor suppresser pathways directly or indirectly by modulating diverse cellular processes, such as metabolism, cell division, differentiation, cell migration, development and apoptosis [5, 8, 14, 24–26].

In HG-SOC, miRNA expression profiles can be associated with distinct clinical features (e.g., tumor subtype, stage, histological grade, metastasis, prognosis, and therapy resistance) [5, 14, 27, 28]. Recently, researchers revealed that aberrantly expressed miRNAs could contribute to the development of clinically-relevant molecular classification system of histologically same type ovarian cancer (HG-EOC) and they suggested that the tumor-associated miRNA prognostic signatures might be identified in large enouch patient cohort(s) [5, 13, 14, 27, 28]. The Cancer Genome Atlas (TCGA) consortium has reported their microRNA expression signature derived by cluster analysis using 489 HG-SOC samples [14]. However, even using large dataset, TCGA classifier exhibited a weak concordance between the molecular subtypes (predicted via miRNA cluster analysis) and the disease outcomes [8, 14].

For the last decade, several dozen miRNA signatures and putative miRNA biomarkers differencing ovarian cancer and normal ovarian tissue samples or defining the tumor molecular subtypes with clinical relevance and pathways for growth control and cell phenotype have been proposed. However, the clinically relevant miRNA signatures plausible to diagnostics and therapeutics are debated. Currently, there are no assays based on the miRNA signatures applicable for HC-SOC patients’ risk stratification and prognosis.

We still poorly understand the mechanisms of miRNAs synthesis, regulation and interactions in cancers and their integrative effect in multiple oncogenic and tumor suppression pathways. Due to many-to-many interactions between miRNA and mRNA, multiple alterations and feed-back loop control, complex dynamics in both oncogenic and tumor suppressive pathways, simple linear correlation models often does not fit to experimental observations. Indeed, the same miRNA could control either or both oncogenic and tumor suppressive pathways depending on the tumor type, cell status, environmental factors and regulatory pathway contexts [8, 29]. Additionally, taking in to account a high clonal heterogeneity and genome instability of tumors an identification of the biologically important and clinically reproducible miRNA signatures appropriate to the disease classification, diagnostic and personalized prognosis is a great challenge.

The lack of the adequate statistical and computational data-driven methods is also important limitation and is a target for cancer systems biology and oncological bioinformatics. Indeed, treatment of the same massive dataset, using different statistical and computational data-driven methods could lead to different results and conclusions, for example, in an upfront study of bevacizumab in newly diagnosed ovarian cancer [13].

This study develops our feature selection and model-based data-driven prognostic strategy reported in [8]. Our analytical strategy uses the multivariate prognostic algorithm, called the Data Driven Grouping (DDg) method, which provides unbiased and robust selection of small-sized subsets of prognostic variables (miRNAs) and integrates these variables into a prognostic signature. Carried out the survival prediction analysis of HG-EOC patients reported in TCGA database and in other datasets, our method has revealed the 21-miRNA prognostic classifier identified three high-confidence survival-significant subtypes of HG-SOC [8].

Here, the survival prediction strategy is combined with regulatory pathways analysis, leading to the identification of two high confidence, robust and unbiased miRNAs prognostic classifiers, allowing the separation of the HG-SOC patients of a given patients group into three risk subgroups with significantly different 5-year overall survival rates, also correlated with chemotherapy response. We develop so-called miRNA-defined cancer pathway and patient stratification (miRPS) system which integrates several prognostic miRNAs signatures, experimentally confimed target mRNAs and common oncogenic pathways over-expressed in HG-SOC. This system identifies two novel miRNA-based prognostic HG-SOC classifiers and predicts the links of the members of the miRNA prognostic classifiers with the same gene subset common over the eleven signalling pathways important for the HG-SOC malignancy and progression. Two of the 11 pathways control the early ovarian follicle development and oocyte maturation processes, suggesting possible tumor cell de-differentiation and aggressive phenotype.

## Methods

### Dataset and pre-processing

The TCGA miRNA dataset was obtained from The Cancer Genome Atlas (TCGA) data portal, which contained 13 batches of 520 samples in total, with 8-47 samples in each batch [14]. Most of the patients were classified as stage III SOC. One tumor miRNA sample for each patient was available. The miRNA expression data were generated using the Agilent Human miRNA Microarray Platform 8X15K, based on the Sanger miRBase (release 10.1). The Agilent oligo 60-mer probes in this platform were produced by SurePrint Technology. Within each batch, quality assessments were first performed to identify poor quality chips and were followed by background correction and normalization using invariant set normalization (ISN) [30]. The data from all batches were combined after batch effect adjustment using the empirical Bayes method (COMBAT) [31]. Before next steps of data analysis, all expression data were rescaled given a log2 base transformation. A fraction of miRNA data were filtered to eliminate those with expression lower than 4.1 and variance of correlation lower than 5. Briefly, these empirical criteria exclude from our analyses a major fraction of non-specific/low-specific miRNA expression signals. We note that the random noise signals are highly enriched in a relatively large fraction of low-expressed miRNA expression signals. They demonstrated a lack of the significant correlations to each other and the specific miRNAs expressed higher than cut-off value 4.1. The last miRNA exhibit a vast majority of significant correlations, which also associated with specific biological functions. Also, we observed that the frequency distribution of miRNA expression signal value for individual tumor samples following the mixture frequency distribution model of two distinct frequency distributions, which could be approximately separated via visual inspection of the frequency histograms or mathematically stronger using numerical parameterization of the mixture of the exponential and the Generalized Pareto probability function (the skewed function with long right tail). The left part of the mixture frequency distribution function of the signal intensity value was enriched with noise /nonspecific miRNA expression signals, which expression values were poor correlated across the samples. Often, they were not detected at confidence level across tumor samples. The (relatively high) expression levels of the miRNAs at the right side of the mixture frequency distribution often correlated to each other and represent the fraction of miRNAs, enriched with specific biological categories and reproducibly detected across tumor samples. We found that cut-off value 4.1 is agreed with the parametrized mixture frequency distribution model. By our estimates, this cut-off value provides ~85% specificity of the signals. Selected hundred sixty-five miRNA genes passed our criteria were utilized as the input data for our workflow analyses.

Shih at al dataset (GEO entry ID: GSE27290) consisting of 62 miRNA data was obtained from advanced SOC patients (stage III and IV) [5]. The miRNA expression dataset was generated using the Agilent Human MicroRNA Microarray Platform 8X15K, V1.0 (beta version of G4470A) based on the Sanger Database 9.1. The Agilent oligo 60-mer probes, used in this platform, were produced by SurePrint Technology. The same pre-processing procedures as the TCGA dataset were applied, and 49 profiles were qualified for this analysis.

### Data-driven grouping and statistically weighting voting groupping methods

DDSS-1D and DDSS-2D are modifications of the one-dimensional (1D) Data-driven grouping (1D-DDg) and two-dimensional (2D) Data-driven grouping methods (2D-DDg) [32–34] (Additional file 1: Methods). DDSS-1D is a computational survival prediction and feature selection method that is based on the fitting of the semi-parametric Cox proportional hazard regression model of patient survival times (*t*) and events (*e*) to gene expression data (*x*). DDSS-D1 (or 1D-DDg) is designed to identify single genes that exhibit a statistically significant influence on patient survival time and two distinct risk subgroups may be found.

According to DDSS-1D method, two possible relationships between the patient risks (lower risk, higher risk) and the expression pattern relatively grouping cut-off value (higher expressed, lower expressed value) are possible. In the case of a parallel pattern, “higher risk – higher expression” or “low risk – low expression”, the relatively higher prognostic gene expression level is associated with the poorer prognosis (a gene exhibits pro-oncogenic behavior). In the case of anti-parallel pattern “higher risk – low expression” or “low risk – high expression”, the relatively higher prognostic gene expression level is associated with better prognosis (a gene exhibits tumor suppressive behavior).

The DDSS-2D (or 2D-DDg) method estimates the optimal partition (cut-off) of the expression level of a gene in a gene pair (Additional file 1:Methods). This method also maximizes the separation of the survival curves related to high- and low-risk of the disease progression/outcome, but this method considers seven distinct expression patterns (combinations) obtained after estimation of the single cut-off the expression value for each gene in a prognostic gene pair [32–34]. The DDSS-2D could be potentially more specific and powerful, but less stable then DDSS-1D.

Commonly, DDSS-1D and DDSS-2D are designed to identify single genes (using 1D-DDg) or gene pairs (using 2D-DDg) that exhibit a statistically significant influence on patient survival and is highly effective when the patient cohort is large enough and proposed to compose two relatively well-populated distinct survivor subgroups. (Additional file 1: Methods).

Statistical Weighting Voting gropping (SVWg) method could overcome these restrictions [8, 34]. Briefly, SWVg (1) uses the results of DDg-derived dichotomization (DDSS-D1 or DDSS-D2) of the patients onto low- and high- risk groups as input data, (2) ranks and selects the most survival significant DDg-derived prognostic variables (e. g., expression values of miRNAs) based on the Wald statistics –log(*P*-value), (3) maximizes the separation of patient cohort onto two, three or more statistically distinct subgroups (specified by the hasard functions), (4) optimizes the number of the prognostic variables (Additional file 1: Methods). The method includes a cross-validation procedure making the results more robust relatively to random errors (Additional file 1: Methods).

### Robust K-means clustering

K-means clustering is performed with *k*=2 using the programs Cluster 3.0 and Java TreeView [35]. The Manhattan distance is utilized, which measures the summation of absolute distances among every dimension (expressed genes and patient samples). Because the results from the K-means algorithm differed among different runs due to the randomly chosen initial clusters, 1000 runs of K-means clustering procedures were performed to assess the stability and quality of the clusters. To get the robustness of clusters, the K-means clustering procedure was repeated 1000 times. The tumor samples, which were always clustered together in all of the 1000 times, and the miRNAs, which were always clustered together in >90% of occasions of the 1000 times, are identified as robust clusters of the patients and utilized in our survival analysis. Kaplan-Meier (K-M) survival analysis was used to calculate the survival status of patients in each cluster and to stratify the patients into a high-risk and low-risk group.

### Gene ontology analysis and identification of pathways that are potentially altered by microRNAs

In this study we used the DIANA Tools, which provide algorithms, databases and software for the annotation of miRNA regulatory elements and their targets to the interpretation of the role of miRNAs in various pathways. DIANA-mirPath v2 and v3 [36] was used to identify molecular pathways that are potentially altered by miRNAs. Gene enrichment analysis of experimentally supported miRNA target genes (representing the target mRNAs encodded by the protein-coding genes) were obtained from DIANA-TarBase v6 (and also v7). We used a significant cut-off value of *P*<0.05. The predicted miRNA target gene lists (encodining the miRNA trarget mRNAs) were generated by the DIANA-microT-CDS algorithm with a significance level of *P*<0.0001. We screened the protein subsets within known KEGG pathways database for the correspondiung mRNAs potentially targeting by the miRNAs studied in this work. Because of a large number of predictive target genes for the 137 miRNAs of the K-means analysis miRNA signature, in our pathway analyses we used only experimentally supported target genes. Gene ontology analyses were performed via DAVID Bioinformatics 6.7 tools [37] and MetaCore™ software (version 6.8 build 29806, from GeneGo, Inc.).

### Multivariate analysis and kappa test of association

The simultaneous prognostic effect of various factors was determined in a multivariate analysis using Cox proportional-hazards model. We evaluated the level of agreement between the predicted subgroups, and the clinical groups by the weighted kappa correlation measurement using function kappa2 in R package irr [38]. All *P*-values are two-sided.

## Results

### The gene expression and clinical data pre-processing and microarray expression profile re-normalization

**Fig. 1:**
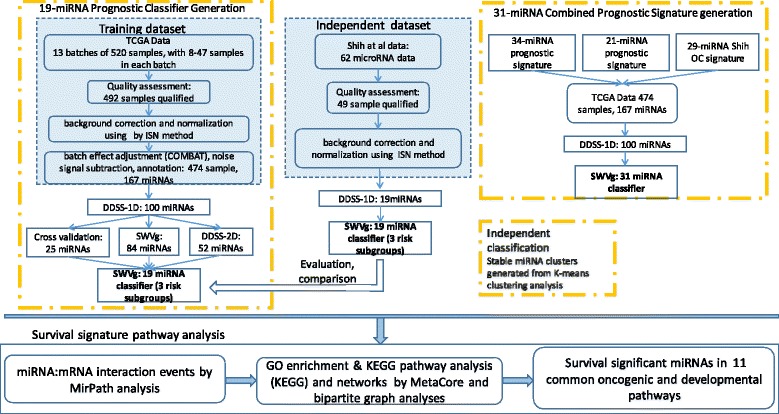
The workflow of the identification of a prognostic signature. The workflow includes the steps of our method of feature selection, cross validation, construction of the prognostic models and regulatory networks. This pipeline processes the miRNA and patient survival data sets, using the updated data-driven grouping (DDg) method, called DDSS. It further performs cluster analysis, statistically weighted voting grouping (SWVg) to select the high-confidence prognostic features (expressed miRNAs) providing the patient stratification two or more disease development risk subgroups. Finally, the work flow links these results with pathway data. DDg and SWVg are the both prognosis and feature selection and patient’s risk classification methods in [32, 34] (see Additional file 1: Methods)

Quality assessment of TCGA miRNA datasets indicated 34 disqualified (28 low-quality chips and six chips without clinical information) and 486 qualified samples. The clinicopathologic characteristics of the 486 qualified patients were summarized in Table 1. Among them, 86% tumors were classified as high histological grades (grade 3 and 4), 90% patients were classified at the advanced stages (stage III or IV) and 80% patients accepted standard chemotherapy treatment. Twelve patients were without survival status data or follow-up information and were excluded from the survival and prognosis analyses. Three probes (representing﻿ miRNA-768-3p, miRNA-801, and miRNA-923) were excluded from our analysis because these probes have no matches in both UCSC browser [39] and miRbase [40] annotations. Finally, the profiles of 474 patients containing 167 miRNAs with expression level higher than background noise were utilized for deriving prognostic miRNA markers (Fig. 1). In the dataset [5], 49 microarray samples passed the qualification assessment and were utilized for the evaluation of our prognostic model developed using the TCGA dataset.

**Table 1:** Clinico-pathologic characteristics of the 486 patients with HG-SOC

Vital status at last follow-up (No. of patients)					
deceased	living			unknown	
262 (54%)	217 (44%)			7 (2%)	
years to death (years)					
min	median			max	
0.02	2.43			12.67	
Years to last follow-up (years)					
min	median			max	
0.02	2.44			15.01	
Years to tumor recurrence (years)					
min	median			max	
0.01	1.23			9.25	
Age at diagnosis					
min	median			max	
27	59			89	
Site of tumor first recurrence (No. of patients)					
loco-regional	metastasis			unknown	
124 (26%)	118 (24%)			244 (50%)	
Stages (No. of patients)					
1		2	3	4	unknown
14 (3%)		21 (4 %)	366 (75%)	72 (15%)	13(3%)
Grade (No. of patients)					
1	2		3	4	unknown
4(1%)	57 (12%)		410 (84%)	1 (0%)	14 (3%)
Tumor residual disease (No. of patients)					
1-10 mm	11-20 mm	>20 mm	no macroscopic disease	unknown	
212 (44%)	26 (5%)	79 (16%)	95 (20%)	74 (15%)	
Primary therapy outcome (No. of patients)					
complete response	partial response	progressive disease	stable disease	unknown	
270 (56%)	56 (12%)	36 (7%)	23 (5%)	101 (21%)

### Combined clustering and differentially expressed miRNAs analysis reveals two ovarian tumor subgroups

**Fig. 2:**
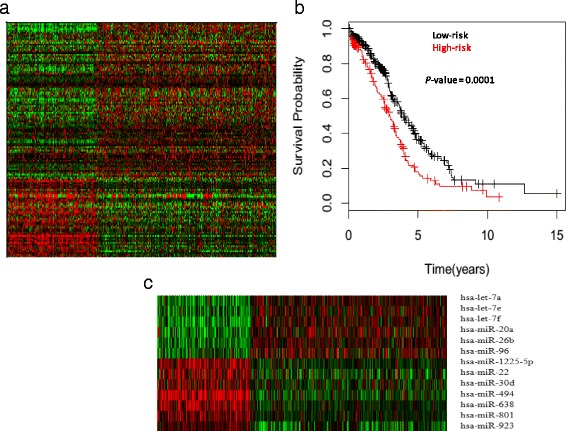
K-means clustering and survival prediction. **a** The K-means clustering analysis of 439 HG-SOC patients using 137 miRNAs. **b** the Kaplan-Meier survival curves of two patient subgroups were constructed from K-means clustering. Survival *P*-value (see Additional file 1: Methods) is shown in the top-right of the survival curves. **c**: 13 survival significant miRNAs are grouped in two clusters associated with the relatively poor and good disease outcome patient subgroups

In total, we investigated the expression profiles of microRNAs in the tumors of 474 HG-SOC patients. Results from robust K-means cluster analysis revealed that 298 samples were always clustered in cluster 1 (low-risk subgroup), and 141 tumor samples were always clustered in cluster 2 (high-risk subgroup). Overall, 93% of the 474 studied samples were always clustered in the one of these groups. Ninety-one miRNAs were clustered together more than 90% of the time in cluster 1, and 46 miRNAs were clustered together more than 90% of the time in cluster 2. The heatmap image of clusters from the selected 137 miRNAs and the 439 tumor samples are presented in Fig. 2a. Among the 137 miRNAs, eight were from the let-7 miRNA family (let-7a, let-7b, let-7d, let-7e, let-7f, let-7g, let-7i and miRNA-98), three were from the miRNA-15-16 family (miRNA-15a, miRNA-15b and miRNA-16), and six were from the miRNA-17 family and its paralog (mir-17, mir-18a, mir-20a, mir-20b, mir-93 and mir-106b). The log-rank *P*-value of the comparison of the Kaplan-Meier curves of two patient clusters was 0.0001 (Fig. 2b).

A set of the experimentally defined targeted protein-coding genes of the 137 miRNAs was strongly enriched (at *P-*value <1E-8) with well-established cancer-associated biological functions and pathways [9–11]. Our GO enrichment analysis suggests that these miRNAs could be involved in direct or indirect control of several common oncogenic pathways, such as p53 signaling, transcriptional mis-regulation in cancer, TGF-beta signaling, PI3K-Akt signaling, MAPK signaling, the Wnt signaling pathway, focal adhesions, adherens junctions, and other pathways. These regulatory pathways play critical roles in cancer stem cells, cancer progression, EMT, metastasis and the drug sensitivity of OC and other cancers. Additionally, several less studied connections of OC miRNAs with the ErbB signaling pathway, osteoclast differentiation, and the chemokine signaling pathway were highly enriched. Interestingly, our top 137 miRNA list does not exhibit strong enrichments of the genes related to cytokine-cytokine receptor interactions, NK cell-mediated cytotoxicity, leukocyte trans-epithelial migration pathways, which were found among survival significant protein-coding genes of EOC samples in other studies [9–11].

Next, we used the Mann-Whitney rank test and a 1.5-fold change cut-off value of the differentially expressed miRNAs which allow us to discriminate relatively strong up-regulated and down-regulated miRNA found in two K-means-derived pateint subgroups.

We observed that expression values of 13 of 137 selected miRNAs differed significantly between the patient subgroups (Fig. 2c, Additional file 2: Tables S1 and S2) (FDR<1E-16). These 13 miRNAs include six down-regulated miRNAs (let-7a, let-7e, let-7f, miRNA-20a, miRNA-26b, miRNA-96) and seven up-regulated miRNAs (miRNA-1225-5p, miRNA-22, miRNA-30d, miRNA-494, miRNA-638, miRNA-801, miRNA-923). Thus, combined clustering and differentially expressed miRNAs analysis reveals two ovarian tumor subgroups with differential survival patterns.

### Top 100 DDg-defined survival significant intra-tumor miRNAs

Detail results of DDSS-1D analysis is presented in Additional file 2: Table S2. TCGA data was used as the training set. The DDSS-1D selects the top 100 survival-significant miRNAs (with a log-rank *P*-value <0.05), separating patients into two risk groups (Additional file 2: Table S2). The prognostic miRNAs show the pro-oncogenic and tumor suppression phenotypes by 50 %. Interestingly, the DDSS-D1-defined 5 most significant prognostic miRNAs show pro-oncogenic phenotype (hsa-miR-638, hsa-miR-483-5p, hsa-miR-222, hsa-miR-1225-5p, hsa-miR-188-5p), while the next most 4 prognostic miRNAs (hsa-miR-148a, hsa-miR-148b, hsa-miR-98, hsa-miR-15b) show tumor-suppressor phenotype.

Additional file 3: Figure S1 shows the examples of the implementation of the data-driven patient grouping methods. Additional file 3: Figure S1A shows the result of DDSS-1D method with a single cut-off value in a random variable range (set of values it can assume). In our example, a random variable is the miRNA-222 expression level in the tumor samples of TCGA patients. One tumor miRNA sample for each patient was available. The method stratifies the HG-SOC patients into relatively low- and high-risk subgroups at Wald statistics *P*-value=2.1E-5. Relatively high expression level of the *miRNA-222* in tumor corresponds to the poor prognosis of the HG-SOC patient. Notice that, oncogenic properties of the miRNA-222 has been experimentally shown, however the survival significance of this miRNA and its expression cut-off value for HG-SOC patients has not been reported. When two similar cut-off values within dynamic range of a single prognostic variable is defined, DDSS-1D method uses 2 similar strongest minima of the log (*P*-value) function for the separation of the patients into three statistically significant prognostic subgroups Additional file 3: Figure S1B shows an example for miRNA-148b predictor. This miRNA exhibits the tumor suppression–like phenotype and separates of the HG-SOC patents on low-, intermediate- and high- risk subgroups.

Additional file 3: Figure S1C shows also a schema of the DDSS-2D method analysis. This method provides patient’s grouping, using one cut-off value for each predictive variable in its domain. The method provides ‘the most significant/optimal’ patient’s grouping (at the smallest Wald statistics *P-*value) for the paired variables (miRNA pairs). The cut-off value for each of the miRNA is optimized via selection the most significant/optimal variant (model) of patient’s grouping. Seven possible grouping models of the paired data within the 2D sample space (2D domain) can be indicated. Additional file 3: Figure S1D shows an example of the expression levels of the miRNA pair (let-7a and mir-130a) which separates of the HG-SOC patients (circles on the left panel) into two subgroups with grouping design 2. This model shows that when the expression values of both miRNAs are higher than corresponding cut-offs of the miRNAs, a relatively low risk subgroup can be isolated. Such kind of the patient grouping could be referred to tumor suppressor-like miRNA phenotype with interaction effect of the miRNAs.

**Fig. 3:**
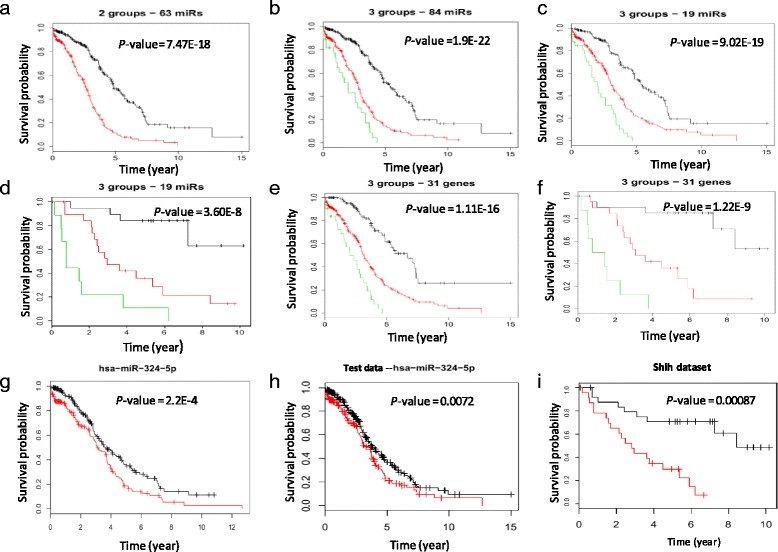
Survival prediction analysis. **a**: K-M survival curves for two SWVg-predicted subgroups (63 miRNAs; TCGA data). **b**: K-M survival curves for three SWVg-predicted subgroups (84 miRNAs; TCGA data). **c** and **d**: the K-M survival curves generated from the 19 miRNA prognostic classifier using TCGA[18] and Shih’s [5]data respectively. **e** and **f**: K-M survival curves for three SWVg-predicted subgroups (31 miRNAs) using TCGA and Shih’s data respectively. **g**, **h** and **i**: K-M survival curves predicted by DDSS-1D based on the expression levels of the miRNA-324-5p in the training (**g**), test (**h**) and independent Shih’s (**i**) datasets. *P*-value (see Additional file 1: Methods) is presented in the top-right of the K-M survival curves. Overall survival (OS) time events were analyzed.

For each patent of a training group (TCGA dataset), the DDSS-1D calculates 100 discrete random variables (Additional file 3: Figure S2). These variables (defined by the 100 survival significant miRNAs), represent a patient by a binary vector which components assign the patient to low-risk or high-risk subgroup. SWVg method used these random vectors as an input dataset for selection of the most statistically significant and robust components of the vector and stratifed the patients onto high-risk or low- risk subgroups (SWVg - DDSS-1D analysis; Methods; Additional file 1: Methods) Additional file 2: Table S2). In TCGA group, SWVg selected the 63 miRNAs which differentiate collectively the patients into two statistically significant prognostic subgroups (low-risk and high-risk subgroups) with the Wald statistics *P*-value of 7.5E-18 (Fig. 3a). This result suggests that the 63 miRNA can be referred to observed tumor heterogeneity and disease outcome. Additional file 2: Table S2 provides prognostic characteristics of the 100 HG-SOC miRNAs derived by SWVg.

Several studies [8, 11, 18] have demonstrated more than two risk significant subgroups in HG-SOC patients. In our study, SWVg method selects 84 prognostic variables and provides a prognostic signature which differentiates the patients onto three statistically distinct subgroups (low-risk, intermediate-risk, and high-risk subgroups) with a small log-rank *P*-value (*P*= 1.9E-22) (Fig. 3b; Additional file 2: Table S2). In this case, the pair-wise K-M function analysis provides the Wald statistics *P*-values as follows: 4.2E-15 for the comparison between low-risk vs. intermediate-risk groups, 2.6E-14 for the comparison between low-risk vs. high-risk groups) and 0.008 for the comparison between intermediate-risk vs. high-risk groups.

Notice that, eleven (let-7a, let-7e, let-7f, miRNA-20a, miRNA-26b, miRNA-96, miRNA-1225-5p, miRNA-22, miRNA-30d, miRNA-494, miRNA-638, miRNA-801, miRNA-923) of the 13 miRNAs reported in our K-means analysis as the miRNA HG-SOC high- and low- risk miRNAs were included in the list of the 100 survival significant miRNA subset (Additional file 2: Table S2) and thus could be considered as the 11-miRNA survival prognostic classifier.

### Prognostic miRNAs selected by the ten–fold cross validation DDSS-D1 analysis

In the ten–fold cross validation DDSS-D1 analysis (Additional file 1: Methods; Additional file 3: Figure S2), we found that 52 miRNAs as statistical significant (*P*-value <0.05) at the >99.7% confidence level. (Additional file 2: Table S3). 25 of these 52 miRNAs had <1% coefficient of variation of cut-off values.

### Prognostic miRNAs selection based on the paired data-driven prognostic analysis: synergistic effects

TCGA miRNA and OS data was used as the training set. DDSS-2D method was applied for selection of the most strong paired predictors separating the pateiens onto low- and high- risk subgroups. We identified 1,656 miRNA pairs with the Wald statistics *P*-value <0.01, and improvement of prognosis outcomes for individual survival-significant miRNAs (Additional file 2: Table S4). 62 most significant miRNAs were appeared in multiple pairs, providing a significant synergistic effect to the stratification of the patients and a better separation of the favorable and unfavorable survival cures than each survival significant miRNA separately.

Notice that, 6 (let-7a, let-7f, let-7e, miRNA-1225-5p, miRNA-638, and miRNA-494) of the 13 miRNAs reported in our K-means analysis as the miRNA HG-SOC classifiers were included in the list of the 62 miRNA subset.

### miRNAs stratifying the TCGA patients into relatively low-, intermediate- and high- risk groups

The Wald statistic -log(*P*-value) defined as the function of the miRNA expression level could show more than one comparable local minimum of the function observed across the patients (Additional file 3: Figure S1B). Using DDSS-1D, we identified 28 miRNAs, which stratify the patients into relatively low-, intermediate- and high- risk groups at the Wald *P*-value<0.05 for pairwise comparisons and at log-rank *P*-value<0.005 for comparison of multiple survival curves (Additional file 2: Table S5). According to miRNA expression values in each stratified subgroups, three prognostic miRNAs could function as the pro-oncogene like factors (miRNA-638, miRNA-572, and miRNA-199a-5p), and eight could function as the tumor suppressor-like factors (miRNA-148b, miRNA-660, miRNA-374a, miRNA-135b, miRNA-574-3p, miRNA-7, miRNA-301a and let-7f) (Additional file 2: Table S5). These results suggest that three (or more) subgroups may be reliably identified when the miRNAs may be combined in a multivariate prognostic signature.

### Identification of the 19-miRNA prognostic signature

To construct a more robust and practically feasible system by clinical perspectives, we derived a small size miRNA prognostic signature as a subset of the 100 survival significant miRNAs, defined DDSS-1D method, which includes a common miRNA subset found by the both DDSS-2D and ten–fold cross validation DDSS-D1 methods (Fig. 1; Table 2; Additional file 2: Table S2). We found that the miRNAs selected by DDSS-2D and the DDSS-1D-CV methods were included in the list of the 84 SWVg-selected miRNAs.

**Table 2:** The 19-miRNA prognostic signature and its characteristics

Signatures miRNA ID	Top 100 survival significant miRNAs selected by DDSS-1D						DDSS-1D ( with 10-fold cross validation)			DDSS-2D
	Log-rank *P*-value	Survival pheno-type^a^	Cut-off	Average expression in subgroup 1	Average expression in subgroup 2	*P-*value in M-W test	*P*-value	Confidence level	CV of cutoff^b^	Frequency of synergistic gene pairs (*P*<0.05)
hsa-miR-483-5p	9.31E-06	2	4.71	4.42	5.05	1.13E-63	3.50E-04	1	0.65%	50
hsa-miR-148a	5.07E-05	1	6.33	6.89	5.84	2.44E-78	6.66E-05	1	0.00%	57
hsa-miR-98	1.49E-04	1	4.70	5.01	4.48	2.50E-70	5.20E-03	1	0.75%	50
**hsa-miR-494**	1.50E-04	2	6.45	5.70	7.23	2.55E-73	8.10E-04	1	0.25%	51
hsa-miR-575	1.82E-04	2	4.91	4.67	5.30	3.01E-77	7.50E-04	1	0.06%	56
hsa-miR-136	3.02E-04	2	4.73	4.40	4.97	7.18E-39	1.40E-02	0.99995	0.48%	53
**hsa-miR-143**	3.33E-04	2	4.99	4.46	5.29	8.21E-28	3.40E-03	0.99993	0.61%	52
hsa-miR-214	9.47E-04	2	5.29	4.68	5.65	1.90E-43	9.10E-03	1	0.35%	59
**hsa-miR-324-3p**	1.36E-03	2	5.79	5.21	6.03	1.25E-20	7.20E-03	1	0.01%	51
**hsa-miR-34a**	2.11E-03	2	5.92	5.59	6.46	7.90E-76	3.90E-03	1	0.03%	54
hsa-miR-377	2.08E-03	2	4.19	4.12	4.57	5.80E-31	2.00E-02	0.99989	0.31%	51
**hsa-miR-10b**	2.76E-03	1	5.59	6.61	5.29	2.58E-58	1.20E-02	0.99996	0.30%	57
**hsa-miR-192**	2.99E-03	1	4.48	4.78	4.34	5.70E-75	3.30E-03	1	0.03%	52
**hsa-miR-134**	3.67E-03	2	5.01	4.53	5.24	9.11E-22	3.30E-03	1	0.11%	54
**hsa-miR-365**	3.85E-03	1	4.95	5.54	4.78	9.72E-27	1.10E-02	1	0.07%	51
**hsa-miR-424**	3.89E-03	1	4.90	5.58	4.69	3.70E-52	2.30E-02	0.99934	0.60%	53
**hsa-miR-205**	7.57E-03	1	4.48	5.75	4.20	1.28E-60	1.20E-02	0.99984	0.06%	59
**hsa-miR-181d**	9.38E-03	1	4.24	4.51	4.18	5.80E-31	1.24E-02	0.99966	0.05%	60

^a^1:: tumor suppressor-like expression pattern 2: pro-oncogenic–like expression miRNA phenotype; defined by the relationships between predicted disease risks and expression level values of the miRNA in cancer tissue (Methods)

^b^CV: coefficient of variation

*Fold change value is calculated as the ratio of the mean expression values of a miRNA of high-risk patients versus that of low-risk patients. 11 novel HG-SOC outcome predictive miRNAs are highlighted in boldface.

The K-M curves from the SWVg of the 19 miRNAs found three patient subgroups that were high significantly different (log-rank *P*-value =9.0E-19; Fig. 3c). Figures 3b and 3c shows that the results of stratification of the TCGA patient’s onto three risk groups providing by the 84 miRNA and 19 miRNA are very similar.

The 19-miRNA prognostic signature subset may be more plausible than the 84 miRNA prognostic set in the context of perspective of assay development and clinical implementation. This 19-miRNA signature includes the following miRNAs: miRNA-10b, miRNA-134, miRNA-136, miRNA-143, miRNA-148a, miRNA-181d, miRNA-192, miRNA-205, miRNA-214, miRNA-324-3p, miRNA-324-5p, miRNA-34a, miRNA-365, miRNA-377, miRNA-424, miRNA-483-5p, miRNA-494, miRNA-575, and miRNA-98. More detailed characteristics of these miRNAs, their features and specific regulatory pathways will be analyzed in the following sections.

### 19-mRNA prognostic signature biological network

The DAVID 6.7 functional annotation tool [37] was utilized to identify gene ontology function clusters of the 19-miRNA prognostic signature subset (Additional file 2: Table S6A). One top-level cluster with an enrichment score value of 15 contained the following gene ontology biological processes terms: cell motion, cell migration, localization of cell and cell motility. Another cluster was a functional category cluster with an enrichment score value of 13 that included apoptosis, anti-apoptosis, and cell death. Therefore, functional and ontology analysis of the mRNA targets indicated that cell motility, cell migration, and apoptosis are critical in ovarian cancer progression.

**Fig. 4:**
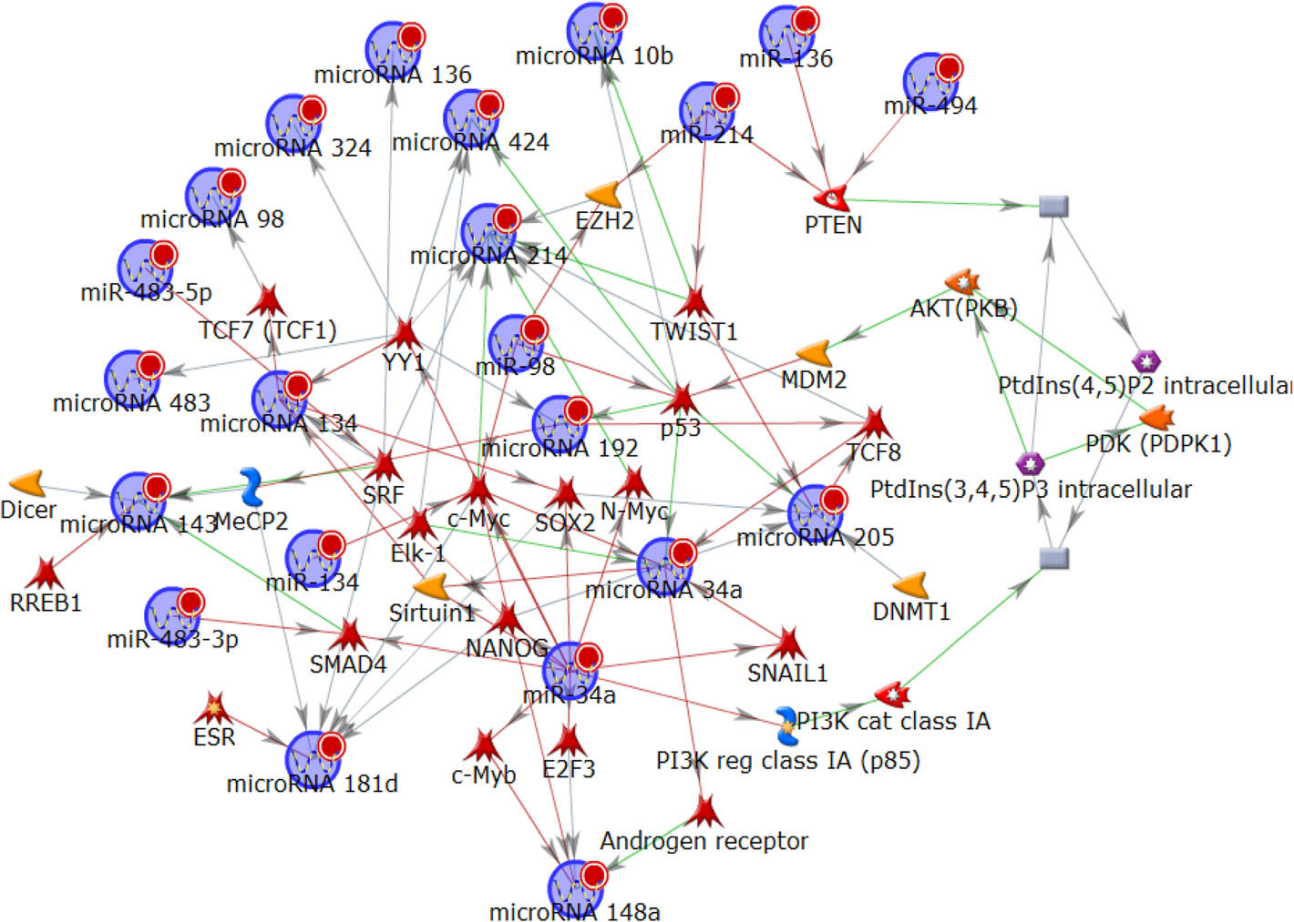
Biological network related to the 19-miRNA prognostic signature. MetaCore software was used for identification of significant biological network of 19-miRNA subset. The detailed legend of the symbols can be found https://portal.genego.com/legends/MetaCoreQuickReferenceGuide.pdf

To develop putative pathway analysis integrating the 19 miRNAs, we applied Dijkstra's shortest paths algorithm, which calculates the shortest directed paths between miRNAs, and found one statistically significant network. This mixture network contains hub proteins c-MYC, p53, YY1, SRF, PTEN, TWIST1, SMAD4, SOX2, NANOG, Elk-1 and TCF8, which play key roles in the stem cell-like properties, initiation and maintenance of the malignancy, tumor suppression, and tumor progression of HG-SOC (Fig. 4). Eight miRNAs, including miRNA-34a, miRNA-214, miRNA-134, miRNA-181d, miRNA-205, miRNA-143, miRNA-148, and miRNA-192, could also serve as multi-targeting small regulatory molecules in the expression of the network. These computational predictions could be experimentally tested and specified in the context of the development of prognostic algorithm and combined pathway-centric, multi-targeting strategies of cancer therapy.

### Association analysis of the 19-miRNA prognostic signature with clinical data and other prognostic signatures

Our appriach demonstrates that TCGA HG-SOC patients can be stratified into three statistically and clinically distinct disease risk groups, using the 19-prognostic miRNA signature, with 5-year overall survival (OS) rate of 51.6%, 20% and 0% for low-, intermediate- and high-risk groups in TCGA dataset, respectively (Table 3).

**Table 3:** Three disease development risk groups derived using the SWVg method.

Signature	Dataset	Low-risk group			Intermediate-risk group			High-risk group		
		% survival	Lower 95% CI	Upper 95% CI	Survival	Lower 95% CI	Upper 95% CI	Survival	Lower 95% CI	Upper 95% CI
19-miRNA signature	TCGA	51.6%	43.0%	61.9%	20.0%	14.6%	27.3%	0.0%	0.0%	0.0%
	GSE27290	84.2%	69.3%	100%	38.1%	20.9%	69.3%	10.0%	1.6%	64.2%
31-miRNA signature	TCGA	62.0%	50.9%	74.7%	23.9%	19.0%	30.7%	0.0%	0.0%	0.0%
	GSE27290	85.0%	70.7%	100%	36.1%	19.70%	66.3%	0.0%	0.0%	0.0%

Five-year overall survival (OS) for the three groups generated using the 19-miRNA and 31-miRNA signatures in ([5]; [14]) datasets

We further evaluated the 19 miRNAs in an independent HG-SOC dataset (Shih et al dataset) [5] and obtained similar results. SWVg identified three patient subgroups with a log-rank *P*- value of 3.6E-8 (Figure 3D), and 84.2%, 38.1% and 10% of 5-year survival rate for the low-, intermediate- and high-risk subgroups respectively (Table 3).

**Fig. 5:**
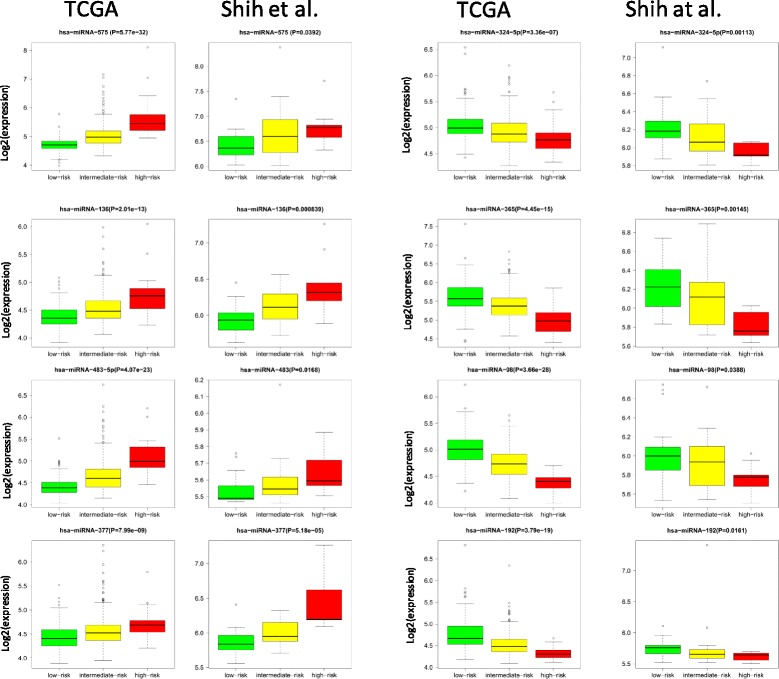
Representative miRNAs that are differentially expressed between the distinct prognostic risks groups

Figure 5 presents the boxplot of expression of eight examples in three groups, including 4 pro-oncogenic miRNAs (miRNA-575, miRNA-136, miRNA-483 and miRNA-377) and 4 tumor suppressor-like miRNAs (miRNA-324-5p, miRNA-365, miRNA-98, and miRNA-192). The miRNAs were independently and strongly significant for survival and may be promising biomarkers for the development prognostic assays and therapeutic implementations.

Chi-square test was used to evaluate the association of categorical clinical indicators (tumor stage, therapy outcome, and tumor residual tissue, etc.) and groupings generated from the 19 miRNAs. Our stratification result was significantly correlated with the patients’ primary therapy outcome (*P*-value=0.0059), stage (*P-*value=0.014) and tumor residue (*P*-value=0.0057) (Additional file 2: Table S7). In dataset [5], the expression levels of 17 miRNAs were found to be survival significant miRNA features (Additional file 2: Table S8). Among these miRNAs, 4 miRNAs were observed in the 19-miRNA prognostic signature identified by SWVg in the TCGA dataset: miRNA-324-5p, miRNA-377, miRNA-181d, and miRNA-34a (Additional file 2: Table S9).

Figures 3g-i present the K-M curves of miRNA-324-5p, demonstrating a tumor suppressor like survival pattern (higher expression in the tumor related to a favorable prognosis; design 2 by DDSS-1D) in the training (TCGA) and independent datasets [5]. This miRNA was reported to inhibit proliferation and enhance differentiation of cancer cells by repressing the transcription factor Gli1 (Gli family zinc finger 1) [41].

In sammary, the results of this section suggest that our DDSS-1D can i) identify potential miRNA biomarkers with high confidence in the cross validation and independent evaluation datasets and ii) SWVg provides a significant cooperative effect on the patient’s stratification via the 19-miRNA prognostic signature.

### Multivariate and pathway analyses

Multivariate analysis using a Cox proportional hazards model demonstrated that the 19-miRNA prognostic predictor outperformed the clinical characteristics, including residual tumor tissue, tumor stage and histologic grade (Table 4). Additionally, Kappa correlation coefficient analysis demonstrated significant associations between patients’ grouping based on our prognostic classification of the patients on high-, intermediate- and low- risk groups and clinical parameters, such as residual tumor size (*P*-value=5E-4), and tumor response to chemotherapy after primary surgery treatment (*P*-value=1E-3) (Table 5). This important finding suggests the potential application of our approach in predicting therapy outcomes. Additionally, we compared the SWVg-derived 19 miRNA-based prognostic patient classification with the reported TCGA grouping where patients were classified based on molecular subtypes, such as “differentiated-type”, “immunoreactive-type”, “mesenchymal-type” and “proliferative-type” [14]. We observed that the SWVg-defined low-risk patients and high-risk patients were significantly correlated with the “proliferative-type” and “the mesenchymal-type”, respectively (*P*-value =4E-18). This analysis suggests that the low risk of the disease progression, defined by the 19-miRNA prognostic classifier, can be associated with sensitivity to post surgery chemotherapy, while the high risk is associated with EMT pathway and, respectively, can be associated with resistance to post-surgery chemotherapy.

**Table 4:** Multivariate Cox proportional hazards analysis of the prognostic 19-miRNA prognostic predictor of HG-SOC patients

Variable	Characteristics	HR	95% CI	*P*-value
SWVg–derived 19 miRNA signature: three groups	low risk group	1		
	intermediate risk group	1.80	1.30-2.50	4.00E-04
	high risk group	5.39	2.68-10.84	2.37E-06
tumor residual disease	1-10 mm	1		
	11-20 mm	0.98	0.53-1.78	0.94
	>20 mm	0.93	0.64-1.36	0.72
	No macroscopic disease	0.50	0.31-0.81	4.78E-03
tumor stage	low (stage I, II)	1		
	high (stage III, IV)	1.87	0.74-4.72	0.19
tumor grade	low (grade 1, 2)	1		
	high (grade 3, 4)	1.41	0.90-2.20	0.14

Note: Results were obtained from the analysis of the HG-SOC TCGA dataset. HR: hazard ratio, CI: confidence interval

**Table 5:** Association between survival patterns predicted by the 19-miRNA prognostic classifier with clinico-pathologic characteristics or molecular subtypes in the TCGA cohort

Characteristic	Subcategory	Low risk		Intermediate risk		High risk		Weighted Kappa	
		(n =231 )		(n =215 )		(n =28)			
		Number	%	Number	%	Number	%	Correlation coefficient	*P*-value^&^
**Age at initial pathologic diagnosis**	age < 50	36	19%	53	20%	4	17%	0.02	0.55
	50 <= age <=70	110	57%	143	55%	8	35%		
	age > 70	42	22%	58	22%	9	39%		
	*others/no information	4	2%	5	2%	2	9%		
**Stage**	Stage I-II	22	11%	13	5%	0	0%	-0.06	0.026
	Stage III	145	76%	203	78%	18	78%		
	Stage IV	25	13%	42	16%	5	22%		
	*others/no information	0	0%	1	0%	0	0%		
**Grade**	Grade 1	30	16%	28	11%	3	13%	-0.03	0.17
	Grade 2	157	82%	228	88%	18	78%		
	Grade 3	1	1%	0	0%	0	0%		
	*others/no information	4	2%	3	1%	2	9%		
**Tumor residual disease**	**No_Macroscopic_disease**	**52**	**27%**	**43**	**17%**	**0**	**0%**	**0.15**	**5.58E-4**
	**1-20 mm**	**83**	**43%**	**144**	**56%**	**11**	**48%**		
	**>20_mm**	**27**	**14%**	**42**	**16%**	**7**	**30%**		
	*others/no information	**30**	**16%**	**30**	**12%**	**5**	**22%**		
**Primary therapy outcome success**	**Complete response**	**130**	**68%**	**133**	**51%**	**5**	**22%**	**0.15**	**1.12E-3**
	**Partial response/stable disease**	**23**	**12%**	**49**	**19%**	**7**	**30%**		
	**Progressive disease**	**11**	**6%**	**23**	**9%**	**1**	**4%**		
	*others/no information	**28**	**15%**	**54**	**21%**	**10**	**43%**		
**^TCGA samples by molecular subtypes**	**Proliferative**	**61**	**32%**	**60**	**23%**	**3**	**13%**	**0.23**	**4.23E-8**
	**Immunoreactive/Differentiated**	**102**	**53%**	**118**	**46%**	**6**	**26%**		
	**Mesenchymal**	**16**	**8%**	**71**	**27%**	**13**	**57%**		
	*others/no information	**13**	**7%**	**10**	**4%**	**1**	**4%**		
**^TCGA samples by miRNA clustering**	C1	54	28%	59	23%	0	0%	-0.07	0.034
	C2	12	6%	89	34%	20	87%		
	C3	113	59%	101	39%	2	9%		
	*others/no information	13	7%	10	4%	1	4%		
**let-7b miRNA Classifier**	**Low Risk**	**119**	**62%**	**40**	**15%**	**0**	**0%**	**0.43**	**<1E-16**
	**Intermediate Risk**	**72**	**38%**	**196**	**76%**	**9**	**39%**		
	**High Risk**	**1**	**1%**	**23**	**9%**	**14**	**61%**		

The measure of agreement was calculated using weighted kappa correlation coefficient, and the significance of the agreement was estimated by kappa2* function in R package *irr*

Note: *These subcategories are not included in the calculation of the Kappa correlation coefficient

^^^Sample groupings were provided by the authors of the TCGA study [14]

^**&**^Significant associations are highlighted in boldface

Notice that, unlike the 19-miRNA classifier, which significantly stratifies patients into three risk-groups, the clustering based on the molecular subtype (mRNA) TCGA signature exhibited the near borderline prognostic significance [8]. Correlation between the 19-miRNA prognostic classifier and TCGA miRNA clustering classes is very week (Table 5). These facts could be explained by the differences in analytical methods.

### Structural uniqueness of the miRNA subset in the 19-miRNA prognostic classifier

**Fig. 6:**
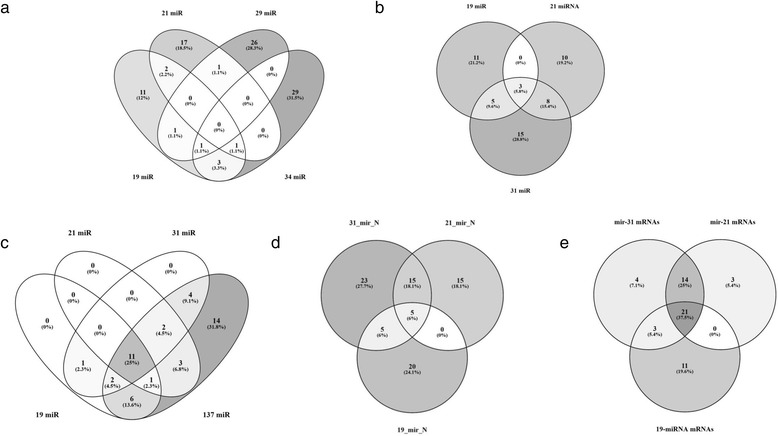
Analysis of the distributions of miRNAs across prognostic signatures, signaling pathways and predicted mRNA targets. **a**: Venn diagram for the miRNA species distributed across 19- miRNA, 21-miRNA, 29-miRNA prognostic signatures and 34-miRNA sets. **b**: Venn diagram for the miRNA species distributed across 19 miRNA, 21 miRNA and 31 miRNA prognostic signature miRNA sets. **c**: Pathway data analysis. Common and unique miRPath-defined signaling pathways associated with the 19-miRNA, 2-miRNA, 31-miRNA and 137 signature sets. The 11 pathways are common miRNA signature's targets. (Table 6; Additional file 2: Table S15). **d**: Venn diagram for the miRNA species referred to the 19-miRNA, 21-miRNA and 31-miRNA prognostic signatures able to control  neurotrophin signaling pathway **e**: Venn diagram for the predicted target mRNAs. These mRNAs are predicted as the targets of the miRNA species referred to the 19-miRNA, 21-miRNA and 31-miRNA prognostic signatures and neurotrophin signaling pathway

The 19-miRNA prognostic signature as a subset of miRNA molecules could be considered as a novel prognostic classifier, because only 3 of the 21 miRNAs were included in our 19-miRNA classifier, 2 miRNAs were included in the 29-miRNA signature in [5], 5 miRNAs were included in the 34-miRNA signature in [42] (Fig. 6a). Additional file 2: Table S10 shows three lists of the miRNA-based survival prediction signatures of HG-SOC and also the list of  81miRNAs providing  the joined set of putative prognostic miRNAs. Interestingly, all miRNA signatures show a few or no common members (Fig. 6a, b), suggesting that miRNA biomarker space is not still far to be complete. In total, the eleven miRNAs were unique in the 19-mRNA prognostic signature (Additional file 2: Table S11).

### Commonness and uniqueness of the survival significant miRNAs across different miRNA signatures

In our previous work [8], we have identified the 21 miRNA-based expression classifier providing a stratification of the HG-SOC patients onto three risk subgroups representing by distinct disease development cancer subtypes: EMT-enriched, mixture (multiple) and cell cycle/mitosis enriched pathways. Importantly, the expression levels of the 21 survival significant miRNAs were correlated with the expression levels of let-7b miRNA and also the mRNAs of the 36-mRNA prognostic classifier of HG-SOC [8].

We found only three common miRNAs in these miRNA subsets. However, in the TCGA dataset a large percentage of the patients (69.4%) (329 out of 474 samples) were assigned to consistent risk subgroups defined by both the 21-miRNA and 19-miRNA prognostic classifiers.

Furthermore, in TCGA [14] and Shih et al [5] datasets 14 miRNAs from the 21-miRNA prognostic classifier and 16 miRNAs from the 19-miRNA prognostic classifier exhibite the same miRNA expression pattern (pro-oncogenic (design 1) or tumor suppressor-like (design 2)) defined in the context of the disease recurrence risks. Thus, these two structurally different miRNA subsets provide similar functional pattern in three subgroup stratification in both Shih et al [5] and TCGA [14] patient groups (Fig. 3, Table 5).

Coinsidence analysis of the 3x3 TCGA patient contingency table of the 19-miRNA and 21-miRNA prognostic classifiers shown strong association between the patient grouping defined by these prognostic signatures (kappa= 0.43, *P*-value<1E-16) (Table 5), suggesting the functional similarity of the target mRNAs and the regulatory pathways.

These findings suggest the commonness of molecular targets and the clinical importance of our signatures.

The 37 miRNAs were unique in the 19-miRNA and 21-miRNA prognostic classifiers (Fig. 6b). Our GO enrichment analysis of the target mRNAs defined by these 37-miRNAs shown a significant enrichment of the gene products referring to the GO categories specified by the 19-miRNA signature target mRNAs.

### 31-miRNA prognostic signature of HG-SOC: A consensus prognostic classifier

Integrating the literature and data driven groping approach, we derived novel prognostic signature. Detail results of the DEG and DDSS-1D or DDSS-2D analyses of TCGA data are presented in Additional file 2: Tables S2 and S4. In TCGA dataset, the 31-miRNA prognostic signature includes a subset of the miRNAs common among the top 100 survival-significant miRNAs and the 81 miRNAs of three published miRNA prognostic signatures (Additional file 2: Tables S2, Table S10 and S11) [5, 8, 42]. Among the 31 miRNAs, 17 miRNAs exhibit the pro-oncogenic and others the tumor suppressor-like prognostic phenotype (Additional file 2: Table S12). Figure 6b shows the Venn diagrams of the miRNAs of the 31-miRNA prognostic signature with miRNAs  of 19-miRNA and   21-miNA signatures. As we expected, the miRNA set of the 31 miRNA prognostic  signature exhibites more common miRNAs in comparision to other miRNA sets. 

Analyzing the 31-miRNA prognostic miRNAs selected by DDSS-1D method, our SWVg selected 23 miRNAs and forms the ‘optimal’ subset of the miRNAs including most statistically significant prognostic variables (expression values), which stratify collectively the TCGA patients into three high-confidence risk subgroups (*P*-value = 1.11E-16; Fig. 3e, Additional file 2: Table S13). Including left 8 DDSS-D -defined RNAs practically does not change Wald statistic p-value.

The 28 of our 31 prognostic miRNAs found in TCGA dataset, were also expressed in Chih et al dataset [5]. A size of the dataset in [5] is relatively small, however using this datasest SWVg selected 10 most significant miRNAs (Additional file 2: Table 13) as an optimal subset of prognostic miRNAs stratifying HG-SOC patents in [5] into three high confidence risk groups (p= 1.22 E-9; Fig. 3f). Importantly, in 9 of the10 cases the prognostic model designs consist of the expression designs found in prognostic miRNAs of TCGA dataset. Thus, our combined 31 miRNA prognostic signature demonstrates a high confidence and reproducible ability to stratify the HG-SOC patients onto three risk  subgroups. We call this classifier  the miRNA consensus prognostic classifier of HG-SOC.

Assuming an important role of miRNA:mRNA interaction networks, we analysed interconnections  between miRNAs  of the 31-miRNA and 19-miRNA signatures with mRNAs of  the 36 mRNA prognostic signature of HG-SOC [8]. The mRNA prognostic signature was selected because this signature has stratified the TCGA HG-SOC patients onto three subgroups and this stratification resulted in the agreement to the 21-miRNA-based prognostic signature [8]. Using DIANA-miR Path v3 database, we identified 10 miRNAs of the 31-miRNA prognostic signature considered as the putative experimentally-supported inhibitors of 9 mRNAs of the 36-mRNA prognostic signature (Additional file 2: Table S14.A). Similarly, we identified 6 miRNAs of the 19-miRNA prognostic signature considered as putative experimentally-supported inhibitors of 17 mRNAs of the 36-mRNA prognostic signature (Additional file 2: Table S14.B). The 7 mRNAs were predicted as putative experimentally-supported targets common in the miRNA sets of the 31-miRNA and 19-miRNA prognostic signatures. Among the miRNAs of the signatures, only one common miRNA (miRNA-148-3p) was linked to the mRNAs (DNMT1, CBX3). We found that multiple co-targeting miRNA pairs represent a common pattern of the miRNA:mRNA interactions (Additional file 2: Table S14). These findings suggest that pro-oncogenic or tumor-suppressor phenotypes of the protein-codding gene expression patterns in the 36-miRNA signature (and probably in other mRNA-based cancer signatures) could be essentially determined by physical interactions of relatively small subset of the miRNAs and controlled by the miRNA regulatory pathways.

### The miRNAs of different prognostic classifiers are co-targeting many mRNAs in common oncogenic- and developmental- associated pathways

DIANA-miRPath tools [36] was utilized to identify the experimentally supported (using Tarbase software) significantly enriched signaling pathways and the computationally predicted (using micro-CDS software) significantly enriched signaling pathways potentially controlled by the miRNA subsets. In particular, using DIANA-miRPath v2, we analyzed the miRNA:mRNA interaction events in four prognostic miRNA subsets

i) the 19-miRNA signature generated in this work,
ii) the 21-miRNA prognostic signature [8],
iii) the subset of 37 non-redundant miRNAs of the 19-miRNA and 21-miRNA prognostic signatures and
iv) the 137-miRNA K-means clustering-defined subset

The results are summarized in Table 6 and Additional file 2: S15. Fig. 6c shows the Venn diagrams for the sets of the significantly enriched signaling pathways targeting by the miRNAs belonging to our 4 miRNA prognostic signatures. TarBase software defined significantly enriched signaling pathways. We also identified 18 common significant signaling pathways predicted by microT-CDS software. The 11 common pathways defined by TarBase and microT-CDS software were included. These eleven common signaling pathways (Figure 6C; Table 6; Additional file 2: Table S15), suggesting functional consistency of the different miRNA subsets (comprising our prognostic signatures) in these signalling pathways. The pathways included nine oncogenic pathways and two ovary developmental pathways. The subset of oncogenic signaling pathways includes: pathways in cancer**,** p53 signaling, transcriptional misregulation in cancer, insulin signaling, TGF-beta signaling, PI3K-Akt signaling, MAPK signaling, cell cycle, and HIF-1 signaling pathways (Table 6). As expected, the pathways in cancer are high enriched by the target mRNAs for the survival significant miRNAs **(**Additional file 2: Table S15). Other, more specific pathways are critical to genome and transcriptome instability, cancer stem cells maintaining, cell cycle, apoptosis, EMT, invasiveness, metastasis and the efficacy of drug therapy [14, 43]. Among these pathways, the cell cycle is a major cancer hallmark regulated by multiple miRNAs in HG-SOC (Additional file 2: Table S15) and MAPK pathway is a well-known pathophysiological module involved in cell proliferation, differentiation, apoptosis and cell migration. Furthermore, our prognostic signatures also identified the neurotrophin signaling and the progesterone-mediated oocyte maturation pathways (Table 6; Additional file 2: Table S15), playing important roles in developmental process, cellular interactions, cell migration, acting reordering, cycle survival, stem cell and neoplastic cell phenotypes.

**Table 6:** Common significant signaling pathways using DIANA-miRPath analysis of miRNAs selected by DDSS, SWVg and K-means methods

KEGG pathway	19 miRNAs		21 miRNAs associated with let-7b [8]		Combination of 19 miRNAs and 21 miRNAs associated with let-7b [8]		31 miRNAs		137 miRNAs from K-means analysis
	Experimental targets	Predicted targets	Experimental targets	Predicted targets	Experimental targets	Predicted targets	Experimental targets	Predicted targets	Experimental targets
Pathways in cancer	1.70E-21	7.16E-15	1.59E-17	4.61E-24	7.80E-25	6.62E-36	1.45E-15	8.05E-62	8.95E-32
p53 signaling pathway	7.41E-12	1.27E-11	2.42E-14	5.02E-21	2.32E-15	1.59E-12	1.33E-19	3.02E-14	5.16E-09
Transcriptional misregulation in cancer	2.36E-11	1.05E-05	0.000165	3.08E-08	4.74E-10	5.12E-12	3.53E-06	2.85E-10	5.42E-19
Insulin signaling pathway	1.52E-05	1.34E-09	0.021307	1.34E-16	3.03E-06	1.13E-20	1.65E-07	3.68E-23	5.48E-13
Neurotrophin signaling pathway	2.84E-05	1.05E-06	0.002062	2.48E-25	4.91E-07	4.26E-19	2.07E-06	1.66E-24	4.17E-12
TGF-beta signaling pathway	0.001486	2.28E-17	1.41E-05	1.49E-20	3.49E-08	2.08E-17	9.88E-09	4.51E-20	6.01E-09
PI3K-Akt signaling pathway	0.002453	1.75E-22	6.96E-05	3.83E-32	0.000515	9.23E-48	6.18E-07	1.74E-54	1.82E-27
MAPK signaling pathway	0.019079	1.34E-09	0.024663	6.89E-23	0.006415	4.21E-37	0.006692	3.18E-44	1.00E-21
Cell cycle	4.54E-38	3.48E-05	1.38E-11	1.64E-03	1.06E-37	7.03E-07	7.97E-18	6.10E-09	2.18E-14
HIF-1 signaling pathway	0.000282	1.37E-04	0.000307	5.47E-07	1.41E-05	8.89E-11	3.6E-09	2.36E-20	9.94E-11
Progesterone-mediated oocyte maturation	0.000609	5.48E-04	0.008724	4.34E-03	2.06E-05	8.33E-07	0.002693	5.87E-14	1.79E-07

Note: Predicted targets were generated by the DIANA-microT-CDS algorithm. The significance level is set at 0.0001

Experimental validated targets were derived from DIANA-TarBase v6.0. The significance level is set at 0.05

### Functional robustness of significant sub-networks associated with 19-miRNA and 31-miRNA prognostic signatures

Network analysis using MetaCore software was also performed to assess the overall functional roles of the 19-miRNA and 31-miRNA prognostic signatures. In this analysis, the miRNAs of these two distinct signatures could form an interactive network with the same target mRNAs. Indeed, canonical pathway analysis predicted that transcription regulation, transcription factors p53, c-Myc and SMAD4 form the network hubs connected with 11 miRNAs of the 19-miRNA prognostic signature (Additional file 3: Figure S4A) and 9 miRNAs of the 31-miRNA prognostic signature (Additional file 3: Figure S4B).

### Reproducibility of the interconnections between miRNAs in the prognostic significant miRNA subsets and the signaling pathways

Next, to analyze a robustness of the identified associations between the prognostic significant miRNA subsets and the downstream signaling pathways, we recapitulated our analysis using the latest version of DIANA-TarBase v.7. This version of the database has been significantly updated with the number of the experimentally supported miRNA functional annotations. The statistical tests have been redesigned and customized. DIANA-miR-Path v.3 database extention including a novel miRNA/gene name suggestion algorithm, enabling the improvement of the functional annotation of miRNA and mRNA combinations. Gene and miRNA annotations were designed from Ensembl (http://www.ensembl.org/index.html ) and miRBase (http://www.mirbase.org/), respectively. DIANA-micro-T-CDS prediction algorithm has also been changed and re-trained. These changes caused multiple variations in the numbers and types of miRNAs and mRNAs, the annotations and the sequence maps (predicted or curated). In this respect, the results based on the most current databases enable a more complete and accurate search at isoform level and better test the reproducibility and robustness of the previous database version predictions. Additionally, DIANA-TarBase v.7 provides many new miRNA:mRNA pairs and their biological characteristics.

To carry out more accurate and complete pathway enrichment analysis, we extended the list of our prognostic miRNA signatures with the miRNAs with nearly identical sequences except for one or two nucleotides (annotated with an additional lower case letter, for example, miR-124a is closely related to miR-124b). The genes that lead to exact identical mature miRNAs but that are located at different places in the genome are usually indicated with an additional dash-number suffix. We also considered the variants in the extended miRNA lists. For example, the pre-miRNAs hsa-mir-148a and hsa-mir-148b lead to an identical mature miRNA (hsa-miR-148) but are from genes located in different genome regions.

Additional file 2: Table S16 includes the updated lists of the miRNAs referring to the 19-miRNA, 31-miRNA prognostic signatures. The table also includes the list of 95 unique miRNA IDs composing all miRNA species included in the updated miRNA sets of the 19-miRNA, 31-miRNA and 21-miRNA prognostic signatures. We found that the most miRNAs are unique for a signature miRNA list. We observed that three miRNAs (hsa-miR-376c-3p, hsa-miR-376c-5p, hsa-miR-103b) did not associated with the target genes/mRNAs (Additional file 2: Table S16). Therefore, we excluded these miRNAs from our further analysis. For seven miRNAs (hsa-miR-134-3p, hsa-miR-134-5p, hsa-miR-181d-3p, hsa-miR-181d-5p, hsa-miR-494-3p, hsa-miR-494-5p, hsa-miR-517c-3p,hsa-miR-98-3p, hsa-miR-98-5p) the target mRNAs were found in micro-T-CDS only. We used micro-T-CDS for specification of the target mRNAs at micro-T statistical threshold value 0.99.

We found that the11 common pathways (Table 6) can be observed in our three updated miRNA datasets of the 19-miRNA, 31-miRNA and 21-miRNA prognostic signature (Additional file 2: Table S16). We found that the MAPK signaling pathway was not significant in the subset referrering to the 21-miRNA or 31-miRNA signatures (Additional file 2: Table S17). The HIF-1 and PI3K-Akt signaling pathways were not significant in the 19-mir signature subset. Using the combine list of 92 miRNAs, DIANA tools selected 10 of the 11 common pathways defined by our previous analysis. MAPK signaling pathway was not significant in this list Additional file 2: Table S17. Figure 6c shows the Venn diagrams charactering the distribution of the number of distinct pathways over 4 prognostic signatures used for identification of the 11 common pathways  controlled by prognostically significant miRNAs.

Interestingly, among many new significant pathways potentially regulating by our miRNA subsets, we obtained strong enrichment of the “oocyte development” pathway, which we found at borderline significant when the DIANA-TarBase v.6 software was applied. Thus, these results support our initial findings and suggestion that at least 11 common signaling pathways could be regulated by multiple miRN:mRNA interactions of a limited number of survival significant miRNAs belonging to the prognostic signatures with different miRNA compositions (Additional file 2: Table S17).

### Multiple miRNAs of the distinct prognostic signatures could target the mRNAs in same pathway

Each significantly enriched KEGG signaling pathway possesses the experimentally supported miRNA:mRNA links, could be formed by the miRNAs of different prognostic signatures. For instance, the nuerotrophin signaling pathway gene list contains 56 distinct encoded for protein genes, which could be the targets of the 83 miRNAs (Additional file 2: Table S18) representing the 19-miRNA, 21-miRNA and 31-miRNA prognostic signatures (30 miRNA species in "19-mirs", 35  miRNA species in  "21_mirs" and 48 miRNA species in "31_mirs" sets). In our statistical analysis of the signalling pathway, we used the list species miRNA presented in Additional file 2: Tables S17-S20 and counted the miRNAs which were at most experimentally supported or at list selected by high level of mirBase TiTG prediction score. Additional file 2: Tables S20-S21 shows detail characteristics of non-uniform distribution of the miRNAs of different signatures across target mRNA/genes and diverse links between miRNAs and their target mRNA/genes. Figure 6d shows the Venn diagrams for the miRNA species referred to the 19-miRNA, 21-miRNA and 31-miRNA prognostic signatures which able to control neurotrophin signaling pathway. The relationships between subsets of the intersected sets indicate that  the major proportion in each set is represented  by the miRNAs uniquely occurred in the prognostic signature. Fig. 6e shows the Venn diagrams for the predicted target mRNAs. By the pathway data analysis, these mRNAs may be consided as experimentally-supported targets of the miRNA species referred to the 19-miRNA, 21-miRNA and 31-miRNA prognostic signatures involving in neurotrophin signaling pathway. A structure of the diagrams suggests that a major fraction of the pathway target genes (38/58) could be potentially regulated by many distinct miRNAs of the two or three miRNA prognostic signatures. Additional file 2: Table S20 and Fig. 6d, e show that a large fraction (21/56) of the 56 genes could be controled by diverse miRNAs belonging to the 19-miRNA, 21-miRNA and 31-miRNA prognostic sets. We observed such preference of  miRNA targeting in other common pathways.

**Fig. 7:**
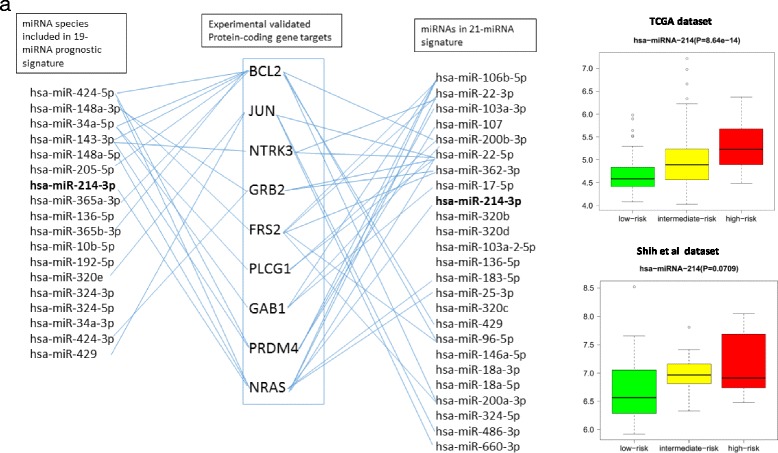
Subset of neurotrophin signaling pathway genes as the target of the survival significant miRNAs representing two subsets of the19-miRNA and 21-miRNA prognostic classifiers. **a** Bipartite graphs link the survival significant miRNAs of the 19-miRNA and 21-miRNA classifiers with their target mRNAs are presented for the 9 representative mRNAs. The experimentally supported and ToTGS prediction score positive mRNAs are included in common 21 mRNA subset shown on Figure 6d. Selected mRNAs are common targets for the miRNAs belonging to the 19-miRNA, 21-miRNA and 31-miRNA classifiers. **b**, **c**: According to our prognostic model,  miRNA-214-3p able to control an expression of multiple target mRNAs resulted in the reproducible pro-oncogenic function in both the TCGA and Shih’s datasets. Details are presented in Additional file 2: Tables S20-S21.

We observed that the number of the potential links between miRNAs and potential target mRNAs is skewed distribution functoion (Additional file 3: Figure S5A,B). The miRNA:RNA links disribution could be visualized with the bipartite graphs. Figure 7a shows two subsets of vertexes representing by the 19-mRNA and 21-miRNAs form the partite sets of two bi-partite graphs centered onto the target mRNAs encoded by protein-coding genes. These figures demonstrate one-to-many, many-to-one and many-to-many regulatory links between miRNAs and targeting mRNAs.

Interestingly, a few miRNAs are also included in the 19-miRNA and 21-miRNA sets. For instance, *mir-214* was included in both signatures. The expression pattern of such miRNAs  are oftern reprodusible. They are differentially expressed across the low-, medium- and high-risk of the disease development groups in both the TCGA [14] and Shih at al [5] datasets, suggesting the pathobiological importance of the miRNAs in HG-SOC outcome (Fig. 7b, c). These findings suggest that a relatively small number of the  miRNAs of the prognostic signatures could target the mRNAs in many HG-SOC regulatory pathway.

Notice that very similar patterns of the miRNA:mRNA interactions we observed in the other statistically significant signaling pathways, for example, TGF-beta, insulin signaling, and HIF-1 signaling pathways (Additional file 2: Tables S15, S17).

Thus, our analysis of mRNA-miRNA bipartite graphs suggests a high plasticity of the miRNA-mediated regulatory network due to one-to-many, many-to-one and many-to-many regulatory links between miRNAs and target mRNAs.

## Discussion

The pipeline leading to specification of the cancer progression stratification system for HG-SOC contains a feature selection procedure with two layers. In the first layer, 100 survival-significant miRNAs were selected by the DDSS-1D method from TCGA dataset. Thirty-one of these 100 miRNAs have been previously reported [5, 8, 42] as survival-significant miRNAs in HG-EOC patients (Fig. 3e-f, Additional file 2: Table S5). This finding suggests a high concordance of our prognostic miRNA subset with joint prognostic miRNA subset derived from previous studies. In the second layer, the three methods (DDSS-1D-CV, SWVg, and DDSS-2D) forms a single module to identify a robust 19-miRNA survival prognostic signature used by SWVg as the HG-SOC prognostic classifier. Several approaches were applied to evaluate the prognostic significance, independence and reproducibility of the 19-miRNA signature.

Eleven common pathways selected by all of 19-miRNA prognostic signature and four other miRNA prognostic signatures suggest high specificity and robustness of the signature in the context of the associated pathways. Importantly, both the 19-miRNA and 21-miRNA prognostic signatures [8] show the significant positive correlations in patient stratification onto three risk subgroups. We found same DDSS-1D grouping design (pro-oncogenic and tumor suppressive like expression patterns) in both the TCGA and Shih et al datasets, supporting our hypothesis that both miRNA signatures can interact with similar target mRNAs and provode similar functions in HG-SOC in very different patient groups.

Variations of nineteen high-confidence prognostic miRNAs were associated with the significant diversity of the development disease risk of HG-SOC patients. Among them, the expressions of *miRNA-136*, *miRNA-214*, and *miRNA-324-5p* were significantly correlated with the expression of *let-7b* and included in the let-7b associated 21-miRNA prognostic signature [8]. Eight miRNAs were selected by previous studies (Additional file 2: Table S12, Additional file 3: Figure S3) [5, 17, 42], and 11 miRNAs can be identified as novel prognostic miRNAs associated with the risk of recurrence of HG-SOC (Table 2). The expression of *miRNA-148a* was associated with post-diagnostic survival [44], *miRNA-483-5p* was included in a signature to differentiate responders from non-responders in ovarian cancer patients [45], and the loss of *miRNA-377* could result in higher proliferation and a shorter survival time [17]. Furthermore, the expression of *miRNA-181d* differentiates cancer, benign and normal ovarian cells [46], *miRNA-205* was overexpressed in ovarian carcinoma [27] and play critical role in the development of miRNA-dependent inhibition of EMT [47], and miRNA-34a could play tumor suppressor role [17]. Additionally, *miRNA-214* was reported to be an anti-apoptotic miRNA associated with high-grade and late-stage tumor progression can regulate survival and drug resistance in ovarian carcinoma. The elevation of miRNA-214 was reported to be responsible for the development of resistance to cisplatin [48]. We propose that these findings could be utilized in future studies of the pathobiological role of these miRNA in HG-SOCs.

The members of the 19-microRNA prognostic classifier, including miRNA-324-5p, miR-377, miR-181d and miR-34a, were predicted to regulate the genes of the nine oncogenic and two early ovary developmental pathways in HG-SOC. These miRNAs might be considered as potential targets for therapeutic intervention of HG-SOC.

In this work, we developed the miRPS method leading to the identification of the eleven HG-SOC-associated pathways, collectively regulated by a small number of survival significant miRNAs belonging to different prognostic signatures. Four different miRNA prognostic signatures supported this finding: (1) the 19-miRNA signature generated in this work, (2) the 21-miRNA prognostic signature [8], (3) the K-means-defined miRNA signature and (4) the 31-miRNA signature (Table 6).

The function enrichment analysis of these signatures indicated that in general cancer-associated miRNAs are highly-enriched in our prognostic signatures. We also found that 9 oncogenic pathways and 2 early ovary developmental pathways could be controlled by miRNAs of the prognostic signatures. The pathways include p53 signaling, transcriptional mis-regulation in cancer, insulin signaling, neurotrophin signaling, TGF-beta signaling, PI3K-AKT signaling, MAPK signaling, cell cycle, HIF-1 signaling, oocyte maturation, essential pathways related to genomic instability, cancer stem cells, cell cycle, apoptosis, invasiveness, metastasis, EMT, reproduction tissue differentiation, neurotrophin signaling and progesterone-mediated oocyte maturation. The gene subsets encoding these mRNAs are highly enriched in the PI3K-AKT signaling pathway, the neurotrophin signaling pathway and the progesterone-mediated oocyte maturation KEGG maps.

Interestingly, via one-to-many, many-to-one and many-to-many miRNA-mRNA and protein-RNA interactions, the eleven miRNA-mediated pathways could be interconnected into more complex tightly connected super-regulatory networks. The use of these networks in conjunction with experimental identification and specialization of the miRNA-mRNA interactions and their functional roles may lead to a better understanding of the integrative and cooperative roles of survival-significant miRNAs in cancer-related protein-coding and non-coding gene pathways and identify rational strategies for post-surgery drug therapy. For instance, the gene expression data demonstrated the interconnection of the IGF1, PI3K, NF-κB, and ERK signaling pathways, which are functionally associated with chemotherapy resistance and the poor treatment response of HG-SOC [49]. Multiple links of the miRNAs of our prognostic signatures with these pathways might be important for the development of miRNA-based treatment strategies of chemo-resistant tumors.

It is important to clarify associations between our new miRNA prognostic signatures and their experimentally validated targets in the known protein coding gene prognostic signatures of HG-SOC. Among the genes of the 36-protein coding gene prognostic signature associated with expression pattern of let7-b miRNA stratifying HG-SOC in three risk subgroups [8], we identified 8 target genes of miRNAs included in the 31-mRNA prognostic classifier and 16 target genes included in the 19-miRNA prognostic classifier (Additional file 2: Table S13). Approximately 47% (17/36) of the protein-coding genes could be collectively targeted by 14 miRNAs found in both miRNA subsets. These findings suggest functional and clinical data associations between the miRNA- and protein- coding gene-based prognostic systems which could be used as a basis for HG-SOC classification and predictive assay development.

It has been reported that some miRNAs and EMT-transformation factors (EMT-TFs) form tightly interconnected negative feedback loops that control epithelial cell plasticity, providing self-reinforcing signals and robustness to maintain epithelial or mesenchymal cell status. Among the most significant feedback loops, the ZEB/miR-200/miR-205 and the SNAIL1/miR-34 networks, which exhibit a clear impact in the regulation of the epithelial or mesenchymal cell phenotype, have been well documented [50]. Of our 19-miRNA prognostic signature, four patient prognostic miRNAs (miR-34, miR-192, miRNA-205, and miRNA-10b) have been reported as members of the EMT pathway [50]. Recent insights into the p53 modulation of the EMT-TF/miRNA loops and epigenetic regulatory mechanisms in the context of metastasis dissemination are also represented by this signature with miRNA-205 and miR-34. Most of these pathways have been discussed in the literature as significant targets of miRNAs in OC tissues or cell lines [50, 51]. However, survival-significant miRNAs, which collectively target the same group of oncogenic pathways, have not been considered as the prognostic factors or perspective therapeutic targets.

Our functional analysis proposed the importance of two early ovary developmental pathways: neurotrophin signaling and progesterone-mediated oocyte maturation (Table 6). Additionally, by mirPath software (using MirBase and microT-CDS tools), KEGG oocyte meiosis pathway is significantly enriched in the most of our survival significant miRNA subsets (data are not presented). According to our knowledge, these pathways have not been considered in the context of pathobiology, patients survival significance and therapeutic targeting of HG-SOC.

In mammals, the neurotrophin family is composed of four members: nerve growth factor (NGF), brain-derived neurotrophic factor (BDNF), neurotrophin-3 (NT3; NTF3), and neurotrophin-4/5 (NT4/5). NGF is the preferred ligand for NTRK1 (TrkA), BDNF and NT4/5 for NTRK2 (TrkB), and NTF3 for NTRK3 (TrkC) [52–54]. The critical importance of NGF, BDNF, NT-4/5, and neurotrophin-3 (NT-3), and their respective TrkA, TrkB, and NTRK receptors in the morphogenesis of ovarian and several other tissues has been shown [52]. Upon neurotrophin-induced stimulation, NTRK receptors can activate the Ras/MAPK pathway, the PI3K (phosphoinositide 3-kinase) pathway, and/or PLC-g1 (phospholipase C gamma 1)-dependent signaling, respectively promoting cell survival, differentiation and activity-dependent neuron plasticity.

The neurotrophin signaling pathway is involved in intra-ovarian cell molecular machinery controlling both the assembly of primordial follicles and the growth of newly formed follicles [52]. The mechanisms underlying the NT3 supportive actions of neurotrophins on these two developmental events have not yet been elucidated. Recent studies suggest that neurotrophins acting via TRKB receptors facilitate early follicle growth by supporting a JAGGED1-NOTCH2 oocyte-to-granulosa cell (GC) communication pathway, which promotes GC proliferation via a c-MYC-dependent mechanism [53].

The roles of neurotrophin receptors in tumor development have been well documented [54–56]. NTRK receptors activation can either support or suppress tumor growth. This is the case for example of *NTRK3*, which is highly expressed in neuroblastomas with good prognosis and highly correlates with patient survival [54]. The emerging evidences of neurotrophins-induced enrichment of cancer stem cells (CSC), which give rise to and maintain the bulk of the tumor are growing [54]. These CSC are thought to be resistant to current chemotherapeutic strategies and thus may provide the principal driving force behind recurrent tumor growth and patient survival [54, 57–60].

Neurotrophin receptors have several isoforms, having different functions regulated by miRNAs. For instance, *NTRK3* has complete and truncated 3’end isoforms. It was shown, that the complete and truncated isoforms of NTRK3 in neuroblastoma cells can be regulated by distinct microRNAs in the isoform-specific manner [54–56]. In this context, *miR-151-3p* is able to specifically regulate the expression of the complete isoform and *miRNA-128*, *mir-9*, *mir-125a* and *mir-125b* are able to regulate the expression of the truncated isoform. Recently, DIANA-TarBase v7.0 has included the experimentally supported interactions of *NTRK3* with *mir-148-3p*, *miR-22-5p*, *miR-22-3p*, *miR-488*, *miR-324-5p*. Interestingly, that *mir-125a-5p* is survival significant miRNA included in the 100 DDSS-1D-defined significant miRNAs. Additionally, *mir-148-3p*, *miR-22-5p*, *miR-22-3p* are included in our 19-miRNA, 31-miRNA, 21-miRNA HG-SOC survival classifiers. The progesterone-mediated oocyte maturation pathway is connected with the PI3K pathway and, according to our results, is also regulated by the miRNAs common for the neurotrophin and several other signaling pathways. These results support a hypothesis that a specific subset of the survival significant miRNAs could play biollogically essential and clinically predictable roles in complex regulatory network integrating multiple cancer-related signalling pathways in initiation of milignancy and driving tumor progression. In summary, our study suggests that in HG-SOC the neurotrophin-mediated and progesterone-mediated oocyte maturation pathway(s) post-transcriptionally regulated by survival significant miRNAs could be important in pathogenesis, classification and prognosis of ovarian cancers and the survival significant miRNAs have significant value as the perspective therapeutic targets.

The results of our survival prediction analysis indicate that the patients in low- and high- risk disease development subgroups are significantly correlated with proliferative-like and mesenchymal-like tumors respectively (Table 5). The mesenchymal-like tumors are often characterized by stem cell-like properties, senescence, and chemo-resistance [57–61] and may not respond favorably to treatment [8]. In contrast, the proliferative-like tumors are characterized by fast-dividing cells that could be sensitive to chemotherapy [8, 59–61]. By our classification, the patients with relatively low-risk tumors are more sensitive to standard adjuvant treatment and the 5-year survival rate consist of 51.6-84.2% (Table 3).

Due to the tissue- and cell- specificity and high stability of miRNA in tumors and circulation [50, 51, 57, 62–64] they become critical signals and clinical biomarkers for cancer diagnostic, monitoring, and treatment. Secretory miRNAs are found in apoptotic bodies, micro-vesicles, and exosomes [62–64], which provide an opportunity to develop non-invasive approach for cancer screening, detection and prognosis. The methodology described here could be adapted to computational selection of perspective secretory miRNAs and their target molecules and signaling pathways.

Survival signature and pathway analyses of SOC have been previously conducted [5, 8, 11, 13, 14, 18–20, 65]. However, developing the models which link the outcome predictors for later stage (or high grade) cancers with signaling pathways has been difficult, in part because of data analysis methodological problems (see for references [8, 14, 20, 65, 66]). In [66], the authors have overcome several severe methodological problems. They have used gene expression profiles of a large group of SOC patients (in total 1525 samples) and developed their prognostic model of SOC in the advanced stages. Their meta-analysis has stratified the patients onto relatively low- and high- risk subgroups and used the genes of the 200-gene survival signature to conduct pathway enrichment analysis. However, the signature did not reveal dominant (significant) pathways. In contrast, using several small size miRNA-based prognostic signatures, our analytical strategy (Fig. 1) identified specific and biologically relevant signaling pathways. It led us to the prediction of the significant and previously unknown functional associations between three HG-EOC risk subgroups stratified by different miRNA prognostic classifiers, eleven miRNA-controlled signaling pathways and post-surgery tumor response to primary chemotherapy (Additional file 2: Table S7).

In this study, we have analyzed microarray data allowing carry out many challenging studies. However, technical and biological limitations of the microarrays data exist and currently well-documented. We have also been limited by some available microarray miRNA probe sets and the number patients. In the future, it should be important to extend the number of miRNAs, including splice variance, anti-sense gene' pairs and long ncRNA genes and identify more exhaustive and specific miRNA signatures and context-specific direct miRNA targets, additional oncogenic pathways, and their functional links. Big miRNA and mRNA data, generated by the next generation sequence technologies (e.g., single-cell RNA-seq) and integrating prospective clinical datum may be necessary for further progress in the field. New analytical methods and techniques for the screening the critical miRNA-controllable genes, mRNAs, signaling pathways and noninvasive cell-free miRNAs detection would have a great value.

Thus, our statistically-based biomarker selection and outcome prediction strategy led to results which provide a rational for future more mechanistically-based and clinically-focused studies. Such studies may provide knowledge towards future personalized diagnostic, prognosis and the optimization of therapeutic interventions of HG-SOC. Our HG-SOC classification based on the analysis of multiple prognostic miRNA prognostic signatures and the associated signaling pathways, when further validated, may be useful in individualizing treatment and care for the cancer patients.

## Conclusion

We developed a pipeline for the survival-significant biomarker selection and applied it to HG-SOC. Our big-data analytics methodology identified distinct and reproducible tumor subtypes and automatically selected a relatively small number of intra-tumor miRNAs reflecting patient’s survival subgroups with distinct regulatory pathways and mRNA targets and the post-surgery primary chemotherapy treatment outcomes. The 19-miRNA and 31-miRNA survival predictors exhibited a high performance and a good reproducibility. Our prognostic classifiers concordantly stratified of the HG-SOC patients into three clinical distinct risk subgroups. The low-risk subgroup has a relatively good 5-year survival rate of 51.6-85%, whereas the intermediate- and high-risk groups have 5-year survival rates of 20-38.1% and 0-10% respectively. The miRNA subsets, included in our classifiers, could be involved in the direct mRNA:miRNA interactions in the 11 oncogenic and developmental signaling pathways. The results provide knowledge that could be helpful in our understanding of pathways integrity and complexity mediated by clinically relevant miRNAs and thus could help to predict new molecular targets for diagnostic, prediction and treatment of ovarian cancers. Our results propose the testable biological hypotheses and could facilitate the discovery of key mechanistic components of miRNA-mRNA interactome in HG-SOC and also predict novel clinically relevant prognostic biomarkers and therapeutic strategies leading to rational treatment assignment and improving patient's life quality.

## Additional files

Additional file 1: Methods. Additional Methods. (DOCX 39 kb)

Additional file 2: Table S1. The 13-miRNA subset from K-means. **Table S2.** The 100 survival significant miRNAs and results of the DDSS-1D, DDSS-2D and SWVg analyses. TCGA dataset was analysed. The 10 survival significant miRNAs belonging to the 13 miRNA subset selected by K-means analysis are highlighted. **Table S3.** The 25-miR subset selected by DDSS-1D with ten-fold cross validation. **Table S4.** The frequency of miRs in DDSS-2D miR pairs. The miRNAs which was reported in 13 miRNAs selected by K-means analysis were highlighted in yellow cell. **Table S5.** 28 miRNAs which display the patterns with three groups in DDSS-1D. **Table S6.** Analysis of 19-miRNA prognostic signature. Significant clusters generated from DAVID functional annotation tools. Each DAVID annotation cluster represents one biological theme by grouping similar annotation terms according to the common genes shared by them. **Table S7.** Association analysis of clinical indicators with groups separated by 19 miRNAs from SWV based on DDSS-1D. **Table S8.** Seventeen survival-significant miRNAs in Shih et al signature [5]. **Table S9.** Wald P-values of 4 significant miRNA in TCGA dataset, supported by independent dataset [5]. **Table S10.** Three miRNA survival prediction signatures of HG-SOC. **Table S11. ** Comparison of the 19-miRNA survival prediction signature with other miRNA-based survival prediction signatures of HG-SOC. **Table S12.** Concensus subset of survival significant miRNAs. **Table S13.**  DDSS-1D-based  selection of the 31 miRNAs  and SWVg analysis. **Table S14.**  Interactions between miRNAs and predicted direct  target  mRNAs found  in 36 mRNA HG-EOC prognostic classifier. **Table S15.** Significant signaling pathways (by DIANA-mirPath v.2 software) targeting by the miRNA subsets belonging to five miRNA signatures. The common pathways shared by miRNA signatures from K-means, DDSS-D1, DDSS-D2 and SWVg analyses are marked in boldface. **Table S16.** Initial data lists  for for miRPath-7.3 analysis,  the number target genes and the target identification methods. **Table S17.** Common signaling pathways. **Table S18.** Neurotrophin signalling pathway: Updated lists of the miRNAs refereeing to the 19-miRNA (19_mir_N), 21-miRNA (21_mir_N) and 31miRNA (31_mir_N) prognostic signatures and their characteristics. **Table S19.** Neurotrophin signaling pathway data (miRNAs, mRNA). **Table S20.** Number of miRNAs interacting with a target mRNA associated with gene encoding neurophilin signaling pathway. **Table S21.** Tarbase Experimentally Supported Interactions for miRNAs. (XLS 400 kb)

Additional file 3: Figure S1. Three data-driven patient grouping methods. A: DDSS-1D method with a single cut-off value of a single prognostic variable (*miRNA-222*). It is an example of patient separation into relatively low- and high- risk subgroups; the cut-off value of *miRNA-222* expression levels is defined at a minimum of the Wald statistics log (*P*-value) (left panel) for two K-M functions (right panel). This cut-off value separates patients into statistically significant survival subgroups. High expression level of the miRNA-222 (at cut-off value >5.56) corresponding to the relatively poor prognosis of the patient subgroup (red K-M curve; right panel). B: The DDSS-1D method uses two cut-off values within dynamic range of a single prognostic variable (*miRNA-148b* expression). The method uses 2 similar strongest minima of the log (*P*-value) function (left panel) separating patients into three statistically significant prognostic subgroups (right panel). C: A schema of the DDSS-2D method of patient’s grouping, using one cut-off value for each predictive variable in its domain. The method provides ‘the most significant/optimal’ patient’s grouping (at the smallest Wald statistics *P*-value) for the paired variables (miRNA pairs). The cut-off value for each of the miRNA is optimized via selection of the most significant/optimal variant of patient’s grouping. Seven possible grouping models of the paired data within the 2D domain can be indicated. D: The expression levels of the miRNA pair (let-7a and mir-130a) which separates the HG-SOC patients into two subgroups with grouping design 2. **Figure S2**. Cross validation analysis of the data-driven survival stratification system. Venn diagram analysis of the miRNA from three prognostic models: DDSS-SWV (SWVg, used 84 miRNAs, input data from DDSS-1D; Additional file 2: **Table S2**), DDSS-1D_10CV (DDSS-1D with ten-fold cross validation robustness, 25 miRNAs; Additional file 2: **Table S3**) and DDSS-2D (DDSS-2D, used top 52 miRNAs, having at least 50 synergistic miRNA pairs; Additional file 2: **Table S4**). The subset, which was common across these miRNA sets includes 19 miRNAs. This miRNA subset was used by SWVg to construct the19-miRNA prognostic signature. **Figure S3**. Correlation between two prognostic signatures: 19-miRNA prognostic classifier and 21-miRNA prognostic classifier. **Figure S4.** Significant canonical pathways using the algorithm of transcription regulation that was generated from the 19-miRNA and 31-miRNA prognostic signatures. Pathway data was generated using MetaCore, GeneGo, Inc. The detailed legend of the symbols can be found at https://portal.genego.com/legends/network_legend.html. A: Gene interconnection subnetwork putatively regulated by the miRNAs included into the 19-miRNA prognostic signature. B: Gene interconnection subnetwork formed by the miRNAs included into the 31-miRNA prognostic signature. **Figure S5.** Typical skewed frequency distribution of the number of miRNA:mRNA links. Data for the neurotrophin signaling pathway are presented. (PDF 606 kb)

Additional file 4: Software. R codes. (PDF 194 kb)

## Abbreviations

COMBAT: batch effect adjustment using empirical Bayes method
CSC: cancer stem cells
DAVID: Database for Annotation, Visualization and Integrated Discovery
DDg: data-driven grouping
DDSS: data-driven survival stratification
DDSS-1D-CV: DDSS one dimension cross validation
EMT: epithelial-mesenchymal transition
GC: granulosa cell
HG-SOC: high-grade serous ovarian carcinoma
ISN: invariant set normalization
KEGG: Kyoto encyclopedia of genes and genomes miRNA: microRNA
K-M: Kaplan-Meier
miRPS: miRNA-defined cancer pathway and patient stratification
mRNA: messenger RNA
OC: ovarian cancer(s)
OS: overall survival
SOC: serous ovarian carcinoma
SWVg (DDSS-1D): SWV based on 1D-DDg
SWVg (DDSS-2D): SWV based on 2D-DDg
SWVg: statistically weighted voting grouping
TCGA: The Cancer Genome Atlas
TF: transcription factor
