# Supplementary material for: Identification of common oncogenic and early developmental pathways in the ovarian carcinomas controlling by distinct prognostically significant microRNA subsets
Source: BMC Genomics. 2017 Oct 3;18(Suppl 6):692. doi: 10.1186/s12864-017-4027-5 (PMC5629558; doi:10.1186/s12864-017-4027-5)
Supplement: Supplementary file 1 — Methods. Additional Methods. (DOCX 39 kb) [file 12864_2017_4027_MOESM1_ESM.docx]

**Identification of Common Oncogenic and Early Developmental Pathways in the Ovarian Carcinomas Controlling by Distinct Prognostically Significant MicroRNA Subsets**

**Authors:** Vladimir A. Kuznetsov^1,2*^ Zhiqun Tang^1^, Anna V. Ivshina^1^

**Affiliations:**

^1^Genome and Gene Expression Data Analysis Division, Bioinformatics Institute, A*STAR, Singapore, 138671, Singapore

^2^School of Computer Science and Engineering, Nanyang Technological University, Singapore, 639798, Singapore

***Corresponding author:** Vladimir A. Kuznetsov, PhD, SciD, Division of Genome and Gene Expression Analysis, Bioinformatics Institute, 30 Biopolis Street, #07-01 Matrix, Singapore, 138671

Phone: +65 6478 8288

Fax:+65 64789047

E-mail: [vladimirk@bii.a-star.edu.sg](mailto:vladimirk@bii.a-star.edu.sg)

**Additional file 1: Methods**

**Identification of single survival-significant miRNA in the case that the patient cohort contains two groups: The DDSS-1D method**

DDSS-1D is modified 1D-DDg method described in [1,2]. Let the $M\times N$ matrix $X={(x_{ij})}_{\begin{matrix} i=1,..,M \\ j=1,..,N \end{matrix}}$ denote processed expression data with $N$ miRNAs for $M$ patients. $x_{ij}$ is the expression level of the $j^{th}$ miRNA in the $i^{th}$ patient. Let numeric array $T=(t_{i})$ denote the clinical outcome (survival time) of patients and nominal array $E=(e_{i})$ denote the clinical event (1=deceased, 0=alive). Assuming that the clinical outcomes are negatively correlated with the expression of miRNA $j$*,* patient $i$ can be separated into two groups (1 = “high-risk”, 0 = “low-risk”) at a pre-defined expression cutoff value $c_{j}$ with the following formula:

$y\begin{matrix} j \\ i \end{matrix}=\left\{ \begin{matrix} 1 \left( high-risk \right), if x_{i,j}>c_{j} \\ 0 \left( low-risk \right), if x_{i,j}\leq c_{j} \end{matrix} \right.$
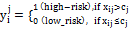
 (1)

The clinical outcome/events can be fitted to the patient groups by a Cox proportional hazard regression model:

$\log h_{i}^{j}(t_{i}\left| y_{i}^{j},\beta^{j} \right)=\alpha^{j}+\beta^{j}{\cdot y}_{i}^{j}$ (2)

where $h_{i}^{j}$is the hazard function, $\alpha^{j}=logh_{i}^{j}(t)$ represents the unspecified log-baseline hazard function when all of the $y$s are zero, and $\beta^{j}$ is the regression parameter and is estimated by using the univariate Cox partial likelihood function

$L\left( \beta^{j} \right)= \prod_{i=1}^{M} \left\{ \frac{exp({\beta^{j}y}_{i}^{j})}{\sum_{k\in R(t_{i})} exp({\beta^{j}y}_{k}^{j})} \right\}^{e_{i}}$ , (3)

where $R\left( t_{i} \right)= \left\{ k: t_{k}\geq t_{i} \right\}$
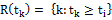
 is the risk set at time $t_{i}$ and $e_{i}$ is the clinical event at the time i.

The Wald statistic (*W*) of $\hat{\beta}^{j}$ is estimated and serves as an indicator of the group discrimination ability for miRNA $j$ at expression cutoff value $c_{j}$. The miRNAs with the largest $\beta^{j}$ Wald Statistics $W_{j}s$ are assumed to exhibit better discriminative ability and thus are called highly survival-significant miRNAs.

Equation (1) suggests that the selection of prognostically significant miRNAs relies on the pre-defined expression cut-off value $c_{j}$ of miRNA $j$ based on which patients can be separated into two groups. Our data-driven “goodness-of-split” method is developed to identify the optimal $c_{j}$ of miRNA $j$ that could most successfully discriminate two groups corresponding to the minimum Wald P value with Wald estimation of $\beta^{j}$. The searching space is among the 10^th^ quantile and 90^th^ quantile of the distribution of the expression values for miRNA $j$ among *M* patients [1,2].

## Cross validation of the DDSS-1D method

Ten-fold cross validation is applied to evaluate the stability and reliability of the models of the DDSS-1D method. The samples are randomly partitioned into 10 subsamples of equal size. On the $k^{th} (k=1,\ldots10)$round
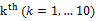
of cross validation, the$k^{th}$ subsample is utilized as test data where the remaining 9 subsamples are combined together and utilized as training data. DDSS-1D analysis is applied to separate the training data into two groups and to derive the intensity cut-off $c_{k_{j},tr}$ and *P*-value of survival curves $p_{k_{j},tr}$ for miRNA $j$. Test data can be separated into two groups by applying the cut-off $c_{k_{j},tr}$. The 10-test grouping results from the 10 rounds of cross validation that can be combined together to produce a single grouping estimation of the whole sample. The Wald statistic for survival $Z$ of the two groups can be computed. The null distribution of $Z$ is generated by permutating the survival time (*T*) and event (*E*) and repeating the entire 10-fold cross validation procedure 500 times. The significance level of this test is evaluated by comparing the grouping results of the cross validation with the null distribution of $Z$. The stability of the intensity cutoff $c_{k_{j},tr}$ for miRNA $j$ is evaluated by calculating the coefficient of variation of $c_{k_{j},tr} (k=1,\ldots10)$
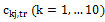
.

## Identification of single survival-significant miRNAs in the case that the patient cohort contains three groups

The notion of data-driven analysis can be extended into samples that may contain more than two groups. Intuitively, two expression cutoffs of a miRNA corresponding to local minimum *P*- values of survival curves on the two deepest valleys could separate samples into three groups.

In this situation, the cut-off and *P* values are obtained in the same way as in the DDSS-1D method via fitting clinical outcomes/events to two patient groups by a Cox proportional hazard regression model. Assuming that the clinical outcomes are negatively correlated with the expression of miRNA $j$, two cut-off values $c_{1j}$ and $c_{2j}$ ($c_{1j}{<c}_{2j}$ ) can be obtained that correspond to the local minimum of two valleys in the curves of log *P*-values when comparing the two groups separated by each cut-off value, and three groups can be found according to following equation:


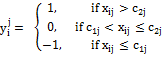
 $y_{i}^{j}= \left\{ \begin{matrix} 1 (h\mathrm{ig}h-risk) & \mathrm{if} x_{ij}>c_{2j} \\ 0 (intermediate-risk) & \mathrm{if} c_{1j}<x_{ij}\leq c_{2j} \\ -1 (low-risk) & \mathrm{if} x_{ij}\leq c_{1j} \end{matrix} \right.$ (4)

Similar calculation procedures as DDSS-1D can be applied. A data-driven “goodness-of-split” method is utilized to identify the optimal $c_{1j}$ and $c_{2j}$ of miRNA $j$ that can most successfully discriminate three groups corresponding to the minimum value of the multiplication of three pairwise Wald *P*-values among three survival curves
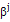
.

## Identification of survival-significant miRNA pairs: The DDSS-2D method

DDSS-2D is modified 1D-DDg method described in [1,2]. DDSS-1D analysis is extended and applied in miRNA pairs when potential synergism exists in two miRNAs. For a given miRNA pair $i, j$ ($i\neq j$), individual optimal cut-offs $c_{i}$ and $c_{j}$ are obtained using the DDSS-1D survival analysis for single miRNAs. According to the cut-offs $c_{i}$ and $c_{j}$ of two miRNAs, patients can be divided into four sections that can be classified into two groups by seven possible designs (distinct variants of 2D partitions), in which one design (design 1) exhibits a correlation effect, 4 designs (design 2 – 5) exhibit a synergetic effect and 2 designs (design 6, 7) exhibit an independent effect. In each design, the clinical information of patients is applied to fit survival curves, and Wald *P*-values for $\beta$
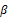
is estimated. Provided that the respective groups’ sample sizes are sufficiently large and that the assumptions of the survival model are satisfied, the best grouping scheme among the seven designs is the one with the smallest *P*-value of the Wald statistics $\beta$ value.

## Statistically weighted voting grouping (SWVg): The identification of multiple synergistic survival-significant miRNAs in the case that the patient cohort contains two groups

The DDSS-1D or DDSS-2D procedure can be utilized to obtain the consensus grouping decision from the grouping information generated by more than two miRNAs [3]. A list of miRNAs is ordered ascendingly according to *P* values generated from DDSS-1D. The numeric grouping value for sample $i$ can be calculated by the formula$G_{i}^{N}=\sum_{j=1}^{N} w_{j}G_{ij}$, where $N$ is the number of distinct miRNAs and $G_{ij}$ is the group allocation for sample $i$ assigned by miRNA$j$ in the DDSS-1D. The weight $w_{j}$ is calculated by the formula

$w_{j}=\frac{-log(p_{j})}{\sum_{m=1}^{N} (-\log\left( p_{m} \right))}$ (5)

where $p_{j}$ is the *P*-value of miRNA $j$ in the DDSS-1D procedure.

In the case that those samples are divided into two groups, patient $i$ can be separated into two groups at a pre-defined cut-off value ($G_{C})$ of$G_{i}^{N}$with the following formula:

$y_{i}^{N}={\{}_{0, \mathrm{if} G_{i}^{N}\leq G_{C}}^{1, \mathrm{if} G_{i}^{N}>G_{C}}$ (6)

Group 1 or 0 could be assigned as either high-risk or low-risk according to the comparison of their survival curves.

A Cox proportional hazard regression model is estimated using the method described in the DDSS-1D procedure. The Wald statistic of $\hat{\beta}^{j}$ is estimated and serves as an indicator to evaluate the ability of group discrimination for miRNA $j$ at cut-off $G_{C}$. The searching space of $G_{C}$ is from 0.2 to 0.8, with an increment of 0.01 for each step. The $G_{C}$which that provides the minimum Wald *P*-values in the searching space is the optimized$G_{C}$. The above-described procedure is repeated for different$N$, which vary from 3 to the number of miRNAs assigned. The number ($N_{opt})$ and combination of $N_{opt}$ miRNAs are optimized for minimum Wald *P*-values.

## Statistically weighted voting grouping (SWVg): The identification of multiple synergistic survival-significant miRNAs in the case that the patient cohort contains three groups

A similar DDSS-1D or DDSS-2D procedure is applied when the samples are divided into three groups [3,4]. Two cut-off values ($G_{C1}, G_{C2}, G_{C1}< G_{C2})$ of$F_{i}^{N}$ are calculated according to the following formula:

$y_{i}^{N}=\left\{ \begin{matrix} 1 & \mathrm{if}G_{i}^{N}>G_{C2} \\ 0 & \mathrm{if}G_{C1}<G_{i}^{N}\leq G_{C2} \\ -1 & \mathrm{if}G_{i}^{N}\leq G_{C1} \end{matrix} \right.$ (7)

Group 1, 0 and -1 are assigned to be high-risk, intermediate-risk or low risk according to the comparison of their survival curves.

A Cox proportional hazard regression model and log-rank statistic estimates are computed. $G_{C_{1}}$ is searched in the range of 0.2 and 0.44, with an increment of 0.01 for each step, whereas $G_{C_{2}}$ is searched in the range 0.56 and 0.8, with an increment of 0.01 for each step. $G_{C_{1}}$, $G_{C_{2}}$ and $N_{opt}$are optimized for the minimum value of log-rank *P*-value of three survival curves.

1. Motakis E, Ivshina AV, Kuznetsov VA: **Data-driven approach to predict survival of cancer patients: estimation of microarray genes' prediction significance by Cox proportional hazard regression model.** *IEEE Eng Med Biol Mag* 2009, **28:**58-66.
2. Grinchuk OV, Motakis E, Kuznetsov VA: **Complex sense-antisense architecture of TNFAIP1/POLDIP2 on 17q11.2 represents a novel transcriptional structural-functional gene module involved in breast cancer progression.** *BMC Genomics* 2010, **11 Suppl 1:**S9.
3. Tang Z, Ow GS, Thiery JP, Ivshina AV, Kuznetsov VA: **Meta-analysis of transcriptome reveals let-7b as an unfavorable prognostic biomarker and predicts molecular and clinical subclasses in high-grade serous ovarian carcinoma.** *International journal of cancer Journal international du cancer* 2013, **134:**306-318.
4. Chen L, Jenjaroenpun P, Pillai AM, Ivshina AV, Ow GS, Efthimios M, Zhiqun T, Tan TZ, Lee SC, Rogers K, Ward JM, Mori S, Adams DJ, Jenkins NA, Copeland NG, Ban KH, Kuznetsov VA, Thiery JP. **Transposon insertional mutagenesis in mice identifies human breast cancer susceptibility genes and signatures for stratification**. *Proc Natl Acad Sci U S A.* 2017 Mar 14;**114**(11):E2215-E2224.
