# Supplementary material for: Identification of common oncogenic and early developmental pathways in the ovarian carcinomas controlling by distinct prognostically significant microRNA subsets
Source: BMC Genomics. 2017 Oct 3;18(Suppl 6):692. doi: 10.1186/s12864-017-4027-5 (PMC5629558; doi:10.1186/s12864-017-4027-5)
Supplement: Supplementary file 3 — Three data-driven patient grouping methods. A: DDSS-1D method with a single cut-off value of a single prognostic variable (miRNA-222). It is an example of patient separation into relatively low- and high- risk subgroups; the cut-off value of miRNA-222 expression levels is defined at a minimum of the Wald statistics log (P-value) (left panel) for two K-M functions (right panel). This cut-off value separates patients into statistically significant survival subgroups. High expression level of the miRNA-222 (at cut-off value >5.56) corresponding to the relatively poor prognosis of the patient subgroup (red K-M curve; right panel). B: The DDSS-1D method uses two cut-off values within dynamic range of a single prognostic variable (miRNA-148b expression). The method uses 2 similar strongest minima of the log (P-value) function (left panel) separating patients into three statistically significant prognostic subgroups (right panel). C: A schema of the DDSS-2D method of patient’s grouping, using one cut-off value for each predictive variable in its domain. The method provides ‘the most significant/optimal’ patient’s grouping (at the smallest Wald statistics P-value) for the paired variables (miRNA pairs). The cut-off value for each of the miRNA is optimized via selection of the most significant/optimal variant of patient’s grouping. Seven possible grouping models of the paired data within the 2D domain can be indicated. D: The expression levels of the miRNA pair (let-7a and mir-130a) which separates the HG-SOC patients into two subgroups with grouping design 2. Figure S2. Cross validation analysis of the data-driven survival stratification system. Venn diagram analysis of the miRNA from three prognostic models: DDSS-SWV (SWVg, used 84 miRNAs, input data from DDSS-1D; Additional file 2: Table S2), DDSS-1D_10CV (DDSS-1D with ten-fold cross validation robustness, 25 miRNAs; Additional file 2: Table S3) and DDSS-2D (DDSS-2D, used top 52 miRNAs, having at least 50 synergistic miRN [file 12864_2017_4027_MOESM3_ESM.pdf]

Figure S1

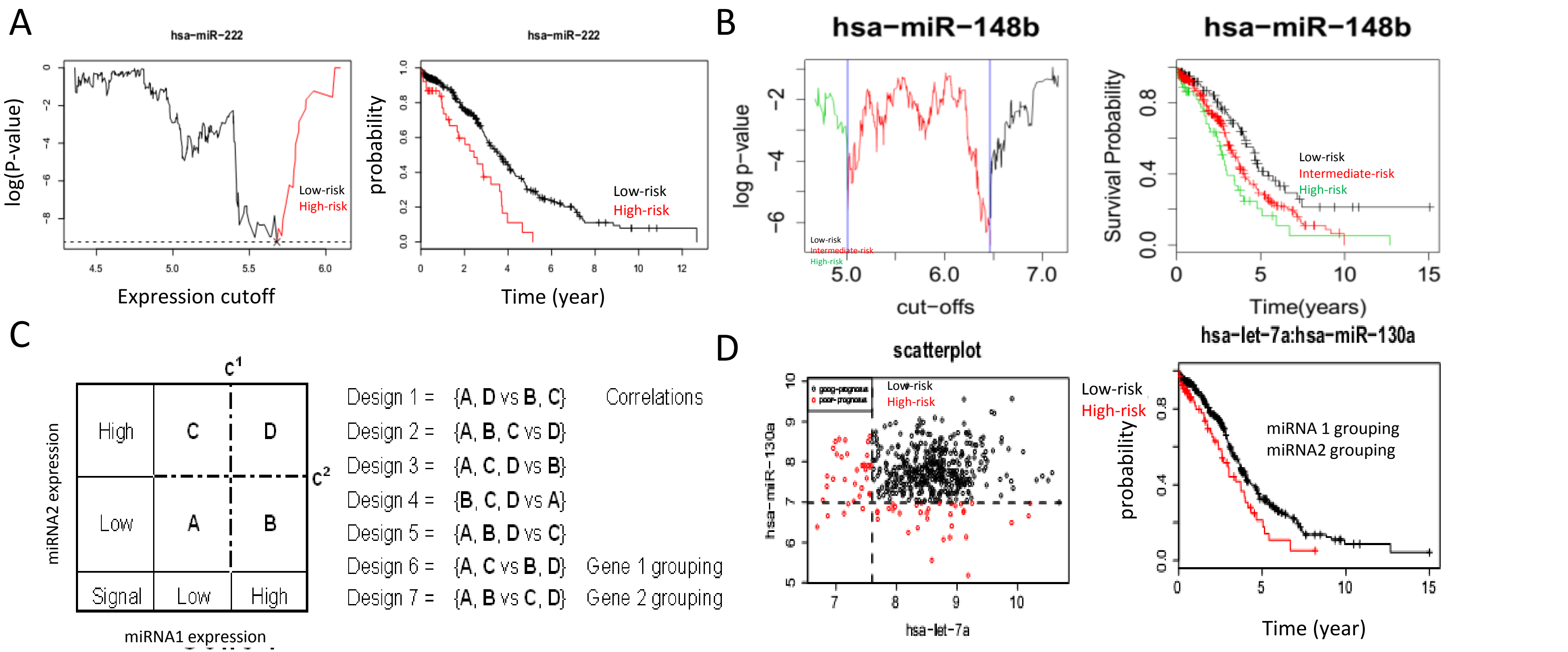
A
hsa-miR-222
log(P-value)
Expression cutoff
Low-risk
High-risk
hsa-miR-222
probability
Time (year)
Low-risk
High-risk
B
hsa-miR-148b
log p-value
cut-offs
hsa-miR-148b
Survival Probability
Time(years)
Low-risk
Intermediate-risk
High-risk
C
miRNA2 expression
miRNA1 expression
High
Low
Signal
Low
High
C
D
A
B
Design 1 = {A, D vs B, C} Correlations
Design 2 = {A, B, C vs D}
Design 3 = {A, C, D vs B}
Design 4 = {B, C, D vs A}
Design 5 = {A, B, D vs C}
Design 6 = {A, C vs B, D} Gene 1 grouping
Design 7 = {A, B vs C, D} Gene 2 grouping
D
scatterplot
hsa-miR-130a
hsa-let-7a
Low-risk
High-risk
hsa-let-7a:hsa-miR-130a
probability
Time (year)
miRNA 1 grouping
miRNA2 grouping

Cross validation for DDSS

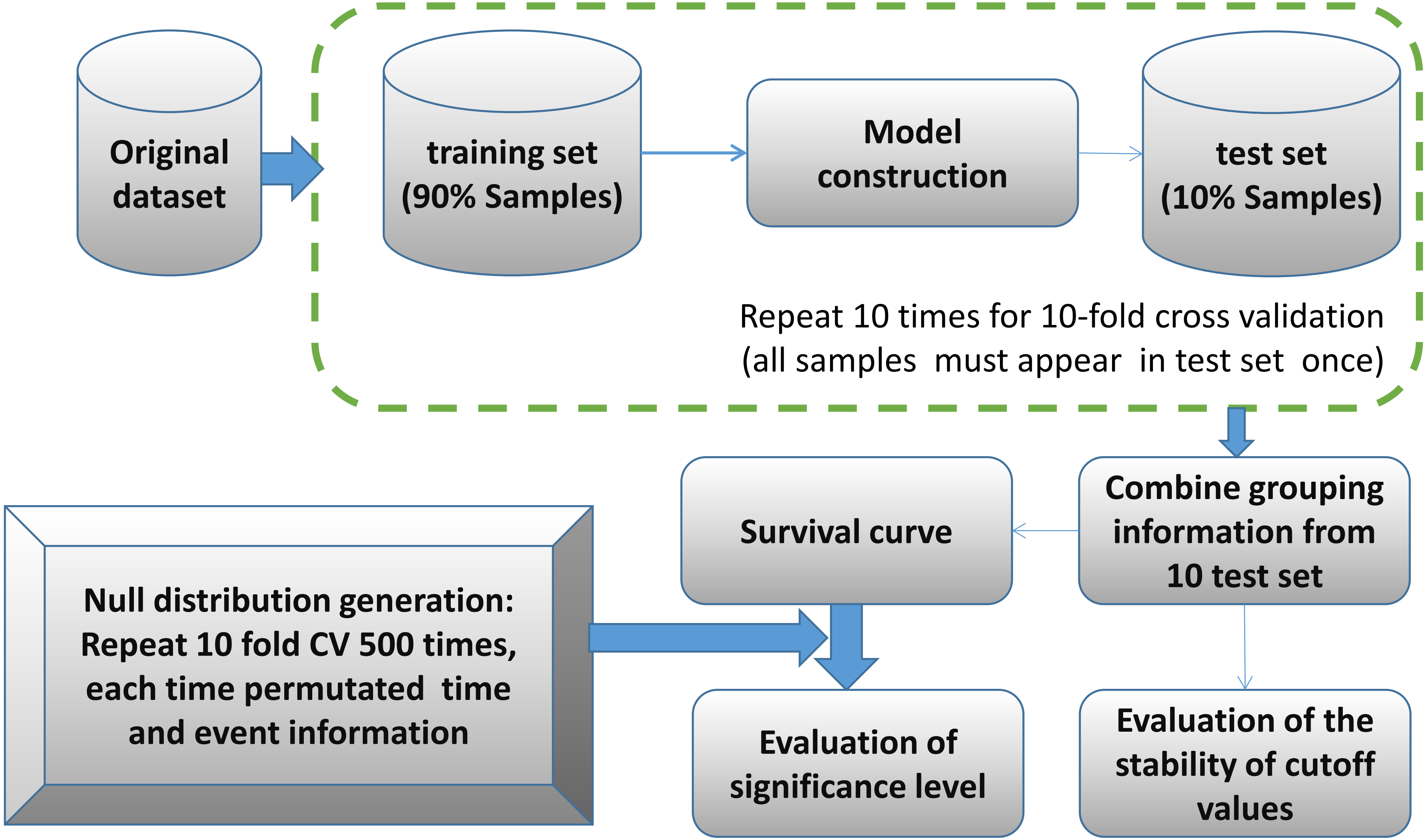

Figure S3

16miRs from 19 miRs with same design in TCGA and Shih at al. data
14miRs from 21miRs with same design in TCGA and Shih at al data

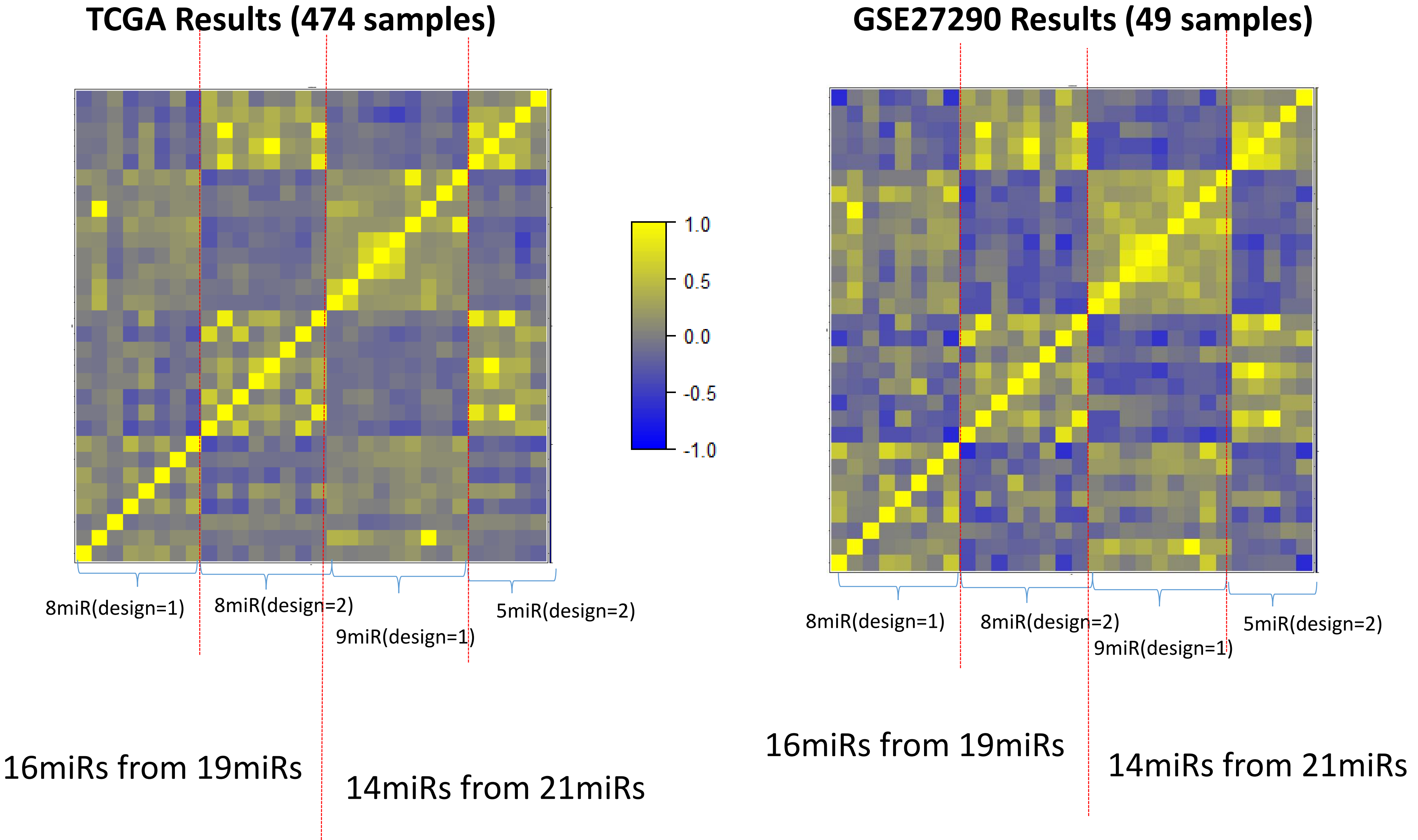

Figure S4

A

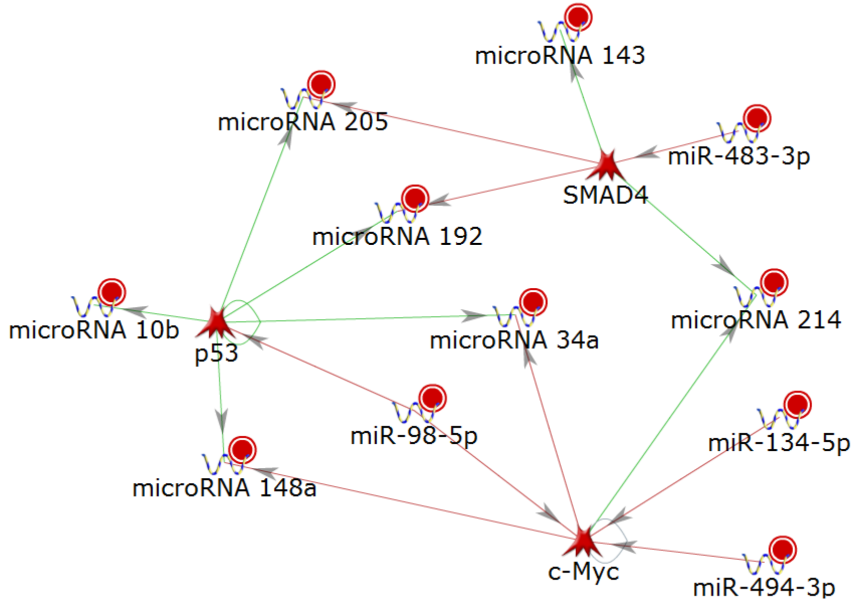

hsa-miR-143
hsa-miR-483
hsa-miR-214
hsa-miR-134
hsa-miR-494
hsa-miR-205
hsa-miR-192
hsa-miR-34a
hsa-miR-98
hsa-miR-148a
hsa-miR-10b

B

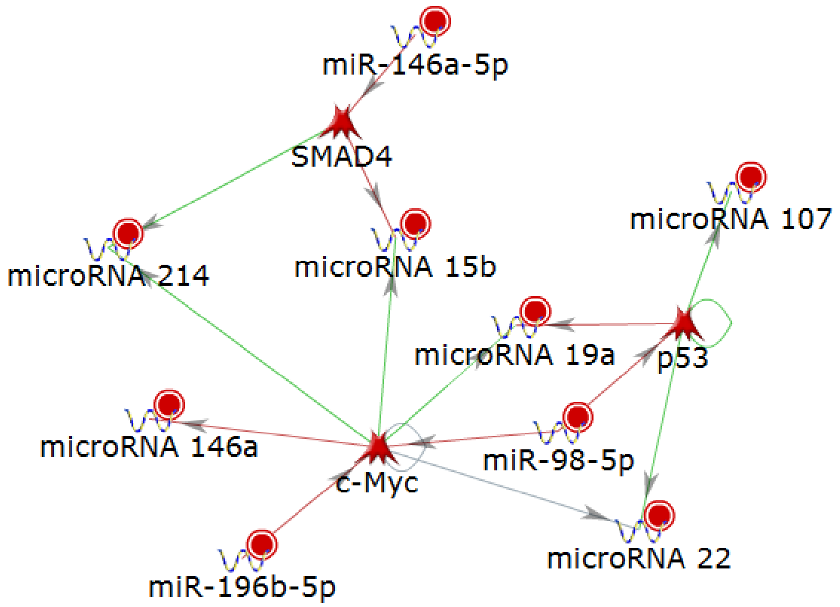

miR-146
miR-19a
miR-15b
miR-214
miR-196b
miR-98
miR-22
miR-146a
miR-107

Figure S5

A

Number of links	Number of target mRNA
1	12
2	7
3	7
4	5
5	8
6	3
7	7
8	2
9	4
11	4
12	4
13	2
14	2
15	1
16	2
20	1
21	1
22	2
24	1
27	1
28	1
30	1
37	2
	80

Frequency distribution of the number of miRNA:mRNA links in Neurotrophic pathway

B

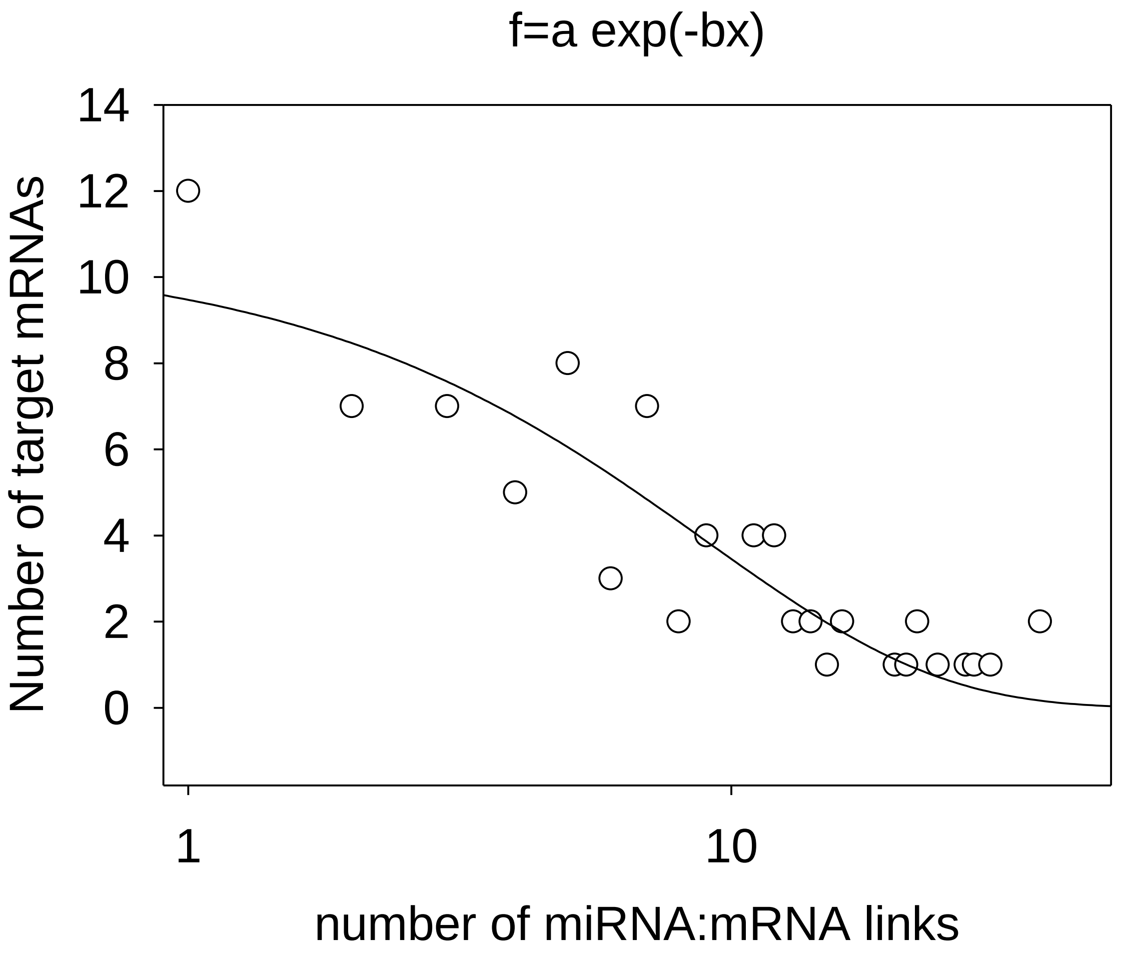

R	Rsqr	Adj Rsqr	Standard Error of Estimate
0.8856	0.7843	0.7741	1.3959

	Coefficient	Std. Error	t	P
a	10.5974	1.1050	9.5904	<0.0001
b	0.1120	0.0175	6.4170	<0.0001
