# Supplementary material for: Identification of common oncogenic and early developmental pathways in the ovarian carcinomas controlling by distinct prognostically significant microRNA subsets
Source: BMC Genomics. 2017 Oct 3;18(Suppl 6):692. doi: 10.1186/s12864-017-4027-5 (PMC5629558; doi:10.1186/s12864-017-4027-5)
Supplement: Supplementary file 4 — Software. R codes. (PDF 194 kb) [file 12864_2017_4027_MOESM4_ESM.pdf]

Identification of common oncogenic and early developmental pathways in the ovarian carcinomas controlling by distinct prognostically significant microRNA subsets

Authors: Vladimir A. Kuznetsov[1,2*] Zhiqun Tang[1], Anna V. Ivshina[1]

Affiliations:

[1]Genome and Gene Expression Data Analysis Division, Bioinformatics Institute, A*STAR, Singapore, 138671
[2]School of Computing Engineering, Nanyang Technological University, Singapore, 639798

***Corresponding author:** Vladimir A. Kuznetsov, Division of Genome and Gene Expression Analysis, Bioinformatics Institute, 30 Biopolis Street, #07-01 Matrix, Singapore, 138671
Phone: +65 6478 8288
Fax:+65 64789047

E-mail: vladimirk@bii.a-star.edu.sg

1D_3grps.r

```
# original written by Makis
# modified by Grace  (updated Jan 19, 2011)
# to separate data into three groups

library(survival)
Data_driven_1DDD_nGrps<-function(genedatum,t,e,patss,title,doplot,
exclude.range=40){   ### ints is vector with patients

  ss<-quantile(genedatum,seq(0,1,0.05))
  range<-genedatum[genedatum>=ss[[2]] & genedatum<=ss[[20]]] # exclude
two end point
  res.pv.alt<-rep(1,length(range))

  group<-matrix(1,length(range),length(t))
  for(j in 1:length(range)){
      for(k in 1:length(t)){
         if(genedatum[k]>range[j]){  group[j,k]<-2   }
      }
        group0<-group[j,]
      if(length(group0[group0==1])>patss &
length(group0[group0==2])>patss){
```

```
          res.pv.alt[j]<- 1-pchisq(survdiff(Surv(t,e)~
as.factor(group0))$chisq,1)
      }
  }

  res.all = cbind(range, res.pv.alt);
  res.all2 = res.all[order(range),]
  opt.pval.index <- find_2minimum(res.all2[,2],  exclude.range=
exclude.range);
  opt.res = res.all2[opt.pval.index, ]  # 1: intensity cuttoff, 2:
pval

  opt.group = rep(3, length(genedatum));
  opt.group[ which (genedatum < min(opt.res[,1]) )]=1 # smallest inten
  opt.group[ which (genedatum > max(opt.res[,1]) )]=2 # largest inten

  opt.range.group = rep(3,dim(res.all2)[1]);
  opt.range.group[ which (res.all2[,1] < min(opt.res[,1]) )]=1 #
smallest inten
  opt.range.group[ which (res.all2[,1] > max(opt.res[,1]) )]=2 #
largest inten


  # logrank test for all 3 groups
  overall.p = 1-
pchisq(survdiff(Surv(t,e)~as.factor(opt.group))$chisq,2)

  #mean survival times

  time1 = mean( t[opt.group==1 & e ==1] );
  time2 = mean( t[opt.group==2 & e ==1] );
  time3 = mean( t[opt.group==3 & e ==1] );
  time = c(time1, time2, time3);

  index = order(time, decreasing=T)
  survival.time = time[index];
  opt.group2=rep(0,length(genedatum));
  opt.group2[opt.group==index[1]] =1    # low_risk
  opt.group2[opt.group==index[2]] =2    # medium_risk
  opt.group2[opt.group==index[3]] =3    # high_risk

  opt.range.group2 = rep(0,dim(res.all2)[1]);
  opt.range.group2[opt.range.group==index[1]] =1    # low_risk
  opt.range.group2[opt.range.group==index[2]] =2    # medium_risk
  opt.range.group2[opt.range.group==index[3]] =3    # high_risk


  npg<-
c(length(opt.group2[opt.group2==1]),length(opt.group2[opt.group2==2]),
length(opt.group2[opt.group2==3]))

 par(mfrow=c(2,2))
```

```
 plot(res.all2[opt.range.group2==1,1],
log(res.all2[opt.range.group2==1,2]),type="l",xlim=c(min(res.all2[,1])
,max(res.all2[,1])),ylim=c(min(log(res.all2[,2])),max(log(res.all2[,2]
))),main=paste(title),xlab="cut-offs",ylab="log p-value", col =1,
cex.axis=1.5, cex.lab=1.5, cex.main=2, lwd=1)
  lines(res.all2[opt.range.group2==2,1],
log(res.all2[opt.range.group2==2,2]), col =2, lwd=1)
  lines(res.all2[opt.range.group2==3,1],
log(res.all2[opt.range.group2==3,2]), col =3, lwd=1)
  abline(v = opt.res[1,1], col=4, pch=22, lty=1, lwd=1);
  abline(v = opt.res[2,1], col=4, pch=22, lty=1,lwd=1);

  plot(survfit(Surv(t, e)~
as.factor(opt.group2)),col=c(1,2,3),main=paste(title),xlab="Time(years
)",ylab="Survival Probability", cex.axis=1.5, cex.lab=1.5,
cex.main=2,lwd=1)

  p12 = 1-
pchisq(survdiff(Surv(t[which(opt.group2!=3)],e[which(opt.group2!=3)])~
as.factor(opt.group2[which(opt.group2!=3)]))$chisq,1);
  p13 = 1-
pchisq(survdiff(Surv(t[which(opt.group2!=2)],e[which(opt.group2!=2)])~
as.factor(opt.group2[which(opt.group2!=2)]))$chisq,1);
  p23 = 1-
pchisq(survdiff(Surv(t[which(opt.group2!=1)],e[which(opt.group2!=1)])~
as.factor(opt.group2[which(opt.group2!=1)]))$chisq,1);
  res1 = c(opt.res[1,1], opt.res[1,2], opt.res[2,1],
opt.res[2,2],overall.p, p12,p13,p23)

#  resall=res1;
#  design=rep(0,length(genedatum));

  return(list(res1,opt.group2,npg, survival.time))
}

find_2minimum <- function (a, exclude.range=40)
# find the index of two minimum
{ b = diff(a);
  c = which(b<0);
  d = sort(a[c+1]);
  id.min1 = which(a ==d[1]) ;
  a1 = a[ -( seq(from=max(1,id.min1-exclude.range), to =
min(id.min1+exclude.range, length(a)), by=1))]
  b1 = diff(a1);
  c1 = which(b1<0);
  d1 = sort(a1[c1+1]);
  id.min2 = which(a ==d1[1]) ;

  return ( c ( id.min1, id.min2 ))
}
```

1D_3grps.r

```
source("1D_3grps.r") # load in functions script

#=================main function=================
#survival time
t<-scan("survivaltime.txt")

#event ( 0=alive, 1=dead )
e<-scan("event.txt")

#read genes and sublist of genes
genes<-as.character(read.table("genes.txt")[[1]])
list1<-as.character(read.table("list1.txt")[[1]])
mm1<-match(list1,genes)

#read the data
data<-t(matrix(scan("data.txt"),nrow=length(t)))
x<-data[mm1,]

#plot title
title<-scan("title.txt",what="character")

resDDp<-matrix(0,length(mm1),8)
#design<-rep(0,length(mm1))
resDDm<-matrix(0,length(mm1),3)
resDDpat<-matrix(0,length(mm1),length(t))
#ptest<-rep(0,length(mm1))
npg<-matrix(0,length(mm1),3)
resTime<-matrix(0,length(mm1),3);


pdf("1DDD_3grps_v1.pdf")
for(q in 1:length(mm1)){
  print (q)
  res<-Data_driven_1DDD_nGrps(x[q,],t,e,20,title[q])
  resDDp[q,]<-res[[1]]
  resDDpat[q,]<-res[[2]]
 # design[q]<-res[[5]]
  resDDm[q,]<-
c(mean(x[q,resDDpat[q,]==1]),mean(x[q,resDDpat[q,]==2]),mean(x[q,resDD
pat[q,]==3]) )
  npg[q,]<-res[[3]]
  resTime =res[[4]]
}
dev.off()

#CUTOFF AND P-VALUES
write(t(resDDp),"res1.txt",ncolumns=8,sep="\t")
```

```
# cutoff_1, P_g1_g23,cutoff_2, P_g12_g3, P_g1_g2_g3, P_g1_g2, P-g1_g3,
P_g2_g3

#patients grouping
write(t(resDDpat),"1DDD_3grps_group.txt",ncolumns=length(t),sep="\t")


#mean intensity for each group
write(t(resDDm),"fold.txt",ncolumns=3,sep="\t")

#number of patients in each group
write(t(npg),"npg.txt",ncolumns=3,sep="\t")

#mean survival time
write(t(resTime),"time.txt",ncolumns=3,sep="\t")
save(list=ls(), file="1DDD_3grps_V2_list1.Rdata")
```

cross_validation_function.r

```
library(survival)
library(marray)
source("/cluster/home/tangzq/R_function/Data_driven_approach/1D.r")

Train_1DDD <- function (data,t,e, gene)
{ mm1       = match(gene, rownames(data));
  x         = as.matrix(data[mm1,]);
  resDDp    =matrix(0,length(mm1),2)
  design    =rep(0,length(mm1))
  intensity =matrix(0,length(mm1),2)
  wilcox    =rep(0,length(mm1))
  group     =matrix(0,length(mm1),length(t))
  colnames(group) = colnames(data)
  proptest  =rep(0,length(mm1))
  n.patient =matrix(0,length(mm1),2)
  n.time    = matrix(0,length(mm1),2)

  for(q in 1:length(mm1))
  { #print (q)
    res     = Data_driven_1DDD(x[q,],t,e,20,title[q],doplot=F)
    resDDp[q,]=res[[1]]
    group[q,] =res[[3]]
    design[q] =res[[5]]
    intensity[q,]=c(mean(x[q,group[q,]==1]),mean(x[q,group[q,]==2]))
    wilcox[q]=wilcox.test(x[q,group[q,]==1],x[q,group[q,]==2])$p.value
    proptest[q] =res[[7]]
    n.patient[q,]=res[[8]]
    n.time[q,]=res[[9]]
  }
  return (data.frame(gene,cutoff = resDDp[,1], pvalue=resDDp[,2], FDR
=p.adjust(resDDp[,2],"BY"), design, intensity=intensity,wilcox,
proptest, n.patient=n.patient, n.time=n.time, group) )
```

```
}

Prediction_1DDD <- function (model = model, test.data, test.e, test.t)
{ gene =  rownames(model);
  mm1  = match(gene, rownames(test.data));
  x    = as.matrix(test.data[mm1,]);
  cutoff = model[,"cutoff"]
  design = model[,"design"]

  group     = matrix(1,length(mm1),length(test.t))
  colnames(group) = colnames(test.data)
  pvalue    = rep(10,length(mm1))
  intensity = matrix(0,length(mm1),2)
  wilcox    = rep(10,length(mm1))
  proptest     = rep(10,length(mm1))
  intensity = matrix(0,length(mm1),2)
  n.patient       = matrix(0,length(mm1),2)
  n.time     = matrix(0,length(mm1),2)

  for(q in 1:length(mm1))
  {# print (q)
    a = x[q,]>cutoff[q]
    if (design[q]==1) { group[q,!a]=2; } else { group[q,a]=2; }
   if( (length(which(group[q,]==1))>1 )&(length(which(group[q,]==2))>1
) )
   {  cox       =  coxph(Surv(test.t,test.e)~ as.factor(group[q,]) );
      pvalue[q] = 1-pchisq(cox$wald.test,1) # wald.test P
      proptest[q]= cox.zph(coxph(Surv(test.t,test.e)
~as.factor(group[q,])))[[1]][3]
      wilcox[q] =
wilcox.test(test.data[q,group[q,]==1],test.data[q,group[q,]==2])$p.val
ue

      n.time[q,] =
c(mean(test.t[(group[q,]==1)&(test.e==1)]),mean(test.t[(group[q,]==2)&
(test.e==1)]) )
      intensity[q,] =
c(mean(test.data[q,(group[q,]==1)]),mean(test.data[q, (group[q,]==2)])
)
    }
    n.patient[q,]   =
c(length(group[q,group[q,]==1]),length(group[q,group[q,]==2]))
  }

  FDR =p.adjust(pvalue,"BY");
  return
(data.frame(gene,cutoff,pvalue,FDR,design,intensity=intensity,wilcox,p
roptest,n.patient=n.patient,n.time=n.time,group) )
}
```

cross_validation_main.r

```
#input: data, t, e
#=====MIR expression data
data = read.table("exp.txt");

id    = as.character(data[1,]);# patient sample ID
id    = id[-1];

gene  = as.character(data[,1])  # gene names
gene  = gene[-(1:3)]

e     = data[2,];   #event
e     = as.numeric(e[-1])
names(e)=id;

t     = data[3,];  #time
t     = as.numeric(as.character(t[-1]) )
names(t)=id;

data  = data[-c(1:3),]   # expression data
data  = data[,-1];
colnames(data)=id;
rownames(data)=gene;

fold =10;     # 10 fold validation

set.seed(1);  # ignore it if you really want random. Set it to check
the result
sam.seq = sample(id, length(id), replace=F)
a = round(length(id)/fold);

topgene = as.character(as.matrix(read.table("genelist.txt"))) ;
topdata =data[topgene,];

save(list=ls(), file="data.Rdata");

# experiment result
res_exp = lapply( c(1:fold), function (i)
{  print (i);
    id0 = c(max(1,(a*(i-1)+1)):min((a*i):length(id)))
    test.id     = sam.seq[id0]
    train.id    = sam.seq[-id0]
    train.data  = as.matrix(topdata[, train.id])
    test.data   = as.matrix(topdata[, test.id] )
    train.e     = e[train.id]
    train.t     = t[train.id]
    test.e      = e[test.id]
```

```
    test.t      = t[test.id]
    # 1DDD partition from training data
    train.Res   = Train_1DDD(train.data,train.t,train.e, topgene)
    model             = cbind( train.Res$cutoff, train.Res$design)
    rownames(model)  = as.character(train.Res$gene);
    colnames(model)  = c("cutoff","design");

    # prediction Result
    test.Res          = Prediction_1DDD(model = model, test.data,
test.e, test.t)
    return (list(i,train.id, test.id, train.Res, test.Res, test.data)
)
 } )

t1_exp = t[res_exp[[1]][[3]] ]
e1_exp = e[res_exp[[1]][[3]] ]
test1_exp=res_exp[[1]][5][[1]][as.character(res_exp[[1]][[4]][,1]),-
c(1:13)]

for (i in c(2:fold))
{ t1_exp = c(t1_exp, t[res_exp[[i]][[3]] ])
  e1_exp = c(e1_exp, e[res_exp[[i]][[3]] ])
  test1_exp=cbind(test1_exp,
res_exp[[i]][5][[1]][as.character(res_exp[[1]][[4]][,1]),-c(1:13)])
}

pdf("grps2_test_all.pdf");
pvalue_grps2_exp    = rep(10,dim(test1_exp)[1])
for ( j in c(1: dim(res_exp[[i]][[5]])[1]) )
{    cox1 =  coxph(Surv(t1_exp, e1_exp)~
as.factor(as.matrix(test1_exp[j,])))
     pvalue_grps2_exp[j] <-1-pchisq(cox1$score,1) # wald.test P
     plot(survfit(Surv(t1_exp, e1_exp)~
as.factor(as.matrix(test1_exp[j,]))),main=paste("test",topgene[j],
":P=",round (pvalue_grps2_exp[j],7)),
col=c(unique(as.numeric(test1_exp[j,]))),xlab="Time(years)",ylab="Surv
ival Probability", cex.axis=1.5, cex.lab=1.5, cex.main=1.5, lwd=1)
}
dev.off()

save(res_exp, file="result_exp.Rdata");

write.table(cbind(topgene, pvalue_grps2_exp),
file="pvalue_exp_topgene.txt", quote=F, sep="\t",
col.names=F,row.names=F)

# bootstraping results......
resample_CV = function (fold, topdata, topgene, id, sam.seq, t, e)
{  res0 = lapply( c(1:fold), function (i)
  {   #print (paste("bootstrap=", k, "fold=",i));
      colnames(topdata) = sample (id, length(id), replace=F)
      id0 = c(max(1,(a*(i-1)+1)):min((a*i):length(id)))
```

```
      test.id     = sam.seq[id0]
      train.id    = sam.seq[-id0]
      train.data  = as.matrix(topdata[, train.id])
      test.data   = as.matrix(topdata[, test.id])
      train.e     = e[train.id]
      train.t     = t[train.id]
      test.e      = e[test.id]
      test.t      = t[test.id]
      # 1DDD partition from training data
      train.Res   = Train_1DDD(train.data,train.t,train.e, topgene)
      model            = cbind( train.Res$cutoff, train.Res$design)
      rownames(model)  = as.character(train.Res$gene);
      colnames(model)  = c("cutoff","design");

      # prediction Result
      test.Res         = Prediction_1DDD(model = model, test.data,
test.e, test.t)
      return (list(i,train.id, test.id, train.Res, test.Res,
test.data) )
   } )

  t1_0 = t[res0[[1]][[3]] ]
  e1_0 = e[res0[[1]][[3]] ]
  test1_0=res0[[1]][5][[1]][as.character(res0[[1]][[4]][,1]),-c(1:13)]

  for (i in c(2:fold))
  { t1_0 = c(t1_0, t[res0[[i]][[3]] ])
    e1_0 = c(e1_0, e[res0[[i]][[3]] ])
    test1_0=cbind(test1_0,
res0[[i]][5][[1]][as.character(res0[[1]][[4]][,1]),-c(1:13)])
  }

  pvalue_grps2_0    = rep(10,dim(test1_0)[1])
  for ( j in c(1: dim(res0[[i]][[5]])[1]) )
  {    cox1 =  coxph(Surv(t1_0, e1_0)~
as.factor(as.matrix(test1_0[j,])))
       pvalue_grps2_0[j] <-1-pchisq(cox1$score,1) # wald.test P
  }

  return (  pvalue_grps2_0 )
}


library(snow);
cl <-  makeCluster(8);      #  stopCluster(cl);

clusterExport(cl,"fold");
clusterExport(cl,"topdata");
clusterExport(cl,"topgene");
clusterExport(cl,"id");
clusterExport(cl,"a");
clusterExport(cl,"sam.seq");
```

```
clusterExport(cl, "e");
clusterExport(cl, "t");
clusterExport(cl, "Train_1DDD");
clusterExport(cl, "Prediction_1DDD");
clusterExport(cl, "resample_CV");
clusterExport(cl, "Data_driven_1DDD");
clusterExport(cl, "newgroups");
clusterExport(cl, "coxph");
clusterExport(cl, "Surv");
clusterExport(cl, "cox.zph");

res.bootstrap <- parLapply(cl, c(1:500), function(i)
{   return( resample_CV(fold, topdata, topgene, id, sam.seq, t,e) )
})

stopCluster(cl)

save(res.bootstrap, file="result_boot.Rdata");


load("result_boot.Rdata");
boot1 = do.call(cbind, res.bootstrap);

write.table(cbind(topgene, boot1), file="pvalue_boot.txt", quote=F,
sep="\t", col.names=F,row.names=F)

#load("pvalue_exp.Rdata")

confidence = rep(0,  length(pvalue_grps2_exp) );
pdf("P_exp_in_boot.pdf");
for ( i in c(1: length(pvalue_grps2_exp) ) )
{  confidence[i] = 1-sum( boot[i,
(boot[i,]<=pvalue_grps2_exp[i])])/sum(boot[i,]);
   plot(density(boot1[i,]),main=paste("bootstrap test:", topgene[i],
"confident level",round (confidence[i],4)), cex.axis=1.5, cex.lab=1.5,
cex.main=1.5, lwd=1, xlim=c(0,1));
  abline (v=pvalue_grps2_exp[i],col="red", lty=2, lwd =3)
}
dev.off();

write.table(cbind(topgene, confidence), file="confidence.txt",
quote=F, sep="\t", col.names=F,row.names=F)
```

4gene.txt

miR-181d

miR-324-5p

miR-34a

miR-377
